# Phenanthroimidazole as molecularly engineered switch for efficient and highly long-lived light-emitting electrochemical cell

**DOI:** 10.1038/s41598-023-29527-7

**Published:** 2023-02-09

**Authors:** Babak Nemati Bideh, Majid Moghadam, Ahmad Sousaraei, Behnoosh Shahpoori Arani

**Affiliations:** 1 Inorganic Chemistry Department, Faculty of Chemistry, Bu-Ali Sina University, Hamedan, Iran; 2 Catalysis Division, Department of Chemistry, University of Isfahan, Isfahan, Iran; 3 Institut Des Matériaux Poreux de Paris, Ecole Normale Superieure, PSL University, 75005 Paris, France; 4 Departamento de Quimica Inorganica Facultad de Ciencias, Universidad Autonoma de Madrid, Madrid, Spain

**Keywords:** Optical materials, Optoelectronic devices and components, Photonic devices

## Abstract

Light-emitting electrochemical cells (LECs) based on Ir(III) complexes owing to the superior advantages exhibit high potential for display and lighting applications. Herein, a series of Ir(III) complexes based on phenanthroimidazole (PI) as an ancillary ligand were synthesized to achieve efficient and highly stable yellow-to-orange LEC devices with fast response. These complexes exhibit appropriate electrochemical stability and significant suppression of concentration quenching in the thin films compared to the archetype complex. The fabricated LECs showed remarkably long device lifetimes over 1400 and 2100 h and external quantum efficiency of 2 and 3% for yellow and orange-LECs, respectively. The obtained t_1/2_ for yellow LEC is much higher than archetype [Ir(ppy)_2_(phen)]^+^ and their phenanthroline-based analogues reported so far. The incorporation of an ionic tethered functional group on PI, improved the mobility of the emissive layer and reduced the device turn-on time by 75–88%. This study shows a facile functionalization and characterization of the PI ligand as well as its potential application in optoelectronic devices (OLED).

## Introduction

Light-emitting electrochemical cells (LECs), are simple, low-cost, and efficient emissive thin-film devices representing great potential for the next generation of optoelectronic devices^1^. These devices exhibit some interesting features that may make them more favourable compared with traditional organic light-emitting diodes (OLEDs), such as a single emissive layer consisting of ionic luminescent species which can be easily fabricated from solution process and their compatibility with an inert-metal cathode (e.g. Ag, Al, and Au) which allows the non-rigorous encapsulation of devices^1–5^. Luminescent materials used as an emitter in LECs generally consist of small molecules, conjugated polymers, ionic transition metal complexes (iTMCs), thermally-activated delayed fluorescence (TADF) molecules, quantum dots, and luminescent perovskite nanoparticles^6–16^. Among them, iTMCs have been received more attention due to their promising advantages. Firstly, unlike organic compounds, the iTMCs are inherently ionic and do not require additional ionic groups. Secondly, for a fluorescent emitter, only 25% of the singlet excitons can be accessible for luminescence, whereas for phosphorescent iTMCs, the triplet relaxation pathway in the form of the radiative deactivation, accelerated through spin–orbit coupling, allows these emitters to reach an internal quantum efficiency (IQE) of up to 100%, which is a requisite to obtain LEC devices with a high external quantum efficiency (EQE). Thirdly, the iTMCs show a stable redox property which is required to achieve a device with high optical stability^17,18^. Phosphorescent cationic cyclometalated iridium (III) complexes are widely exploited iTMCs in optoelectronic applications, thanks to their unique properties such as high phosphorescence quantum yield, chemical inertness, and relatively good photochemical/thermal stability, short and tuneable excited-state lifetimes, as well as high versatility in tuning the emission color through modification of ligand. These favourable characteristics arise from the high ligand-field splitting energies (LFSEs) that are a consequence of the large size of d orbital (5d), high electric charge of the iridium ion (Ir^3+^), and high field strength exerted by anionic cyclometalated ligands (C^N) endowed by strong spin–orbit coupling^18–21^. However, during the first years of iTMC-LEC research, two significant obstacles limited their use in practical applications that are low stability (defined as half-lifetime, t_1/2_, time to reach one-half of the maximum brightness) and long response time (expressed as turn-on time, t_on_: the time that is required to reach a maximum brightness) of devices^22,23^. Several approaches have been developed to increase the device’s lifetimes. The inherent instability of iTMC is attributed to a ligand exchange reaction with the residue of solvent or water molecules during the device operation, forming a non-luminescent complex^14–24,24–26^. Therefore, an efficient way to increase the stability of iTMC can be limiting the access of foreign substances to the metal center. Accordingly, it is indicated that the device stability can be significantly improved by introducing peripheral bulky aromatic groups that increase the complex’s hydrophobicity and prevent water molecules from approaching the metal center. Furthermore, supramolecular-caged which comes from intraligand π-π-stacking of aromatic rings, minimizes the expansion of metal–ligand bonds in the excited state, protecting the ligand exchange by surrounding molecules such as water and solvent^21,27–34^. Another reason for reducing the lifetime of iTMC-LECs is their operational mechanism. During the operation of LECs, the n- and p-doped regions grow continuously adjacent to the electrodes. Because of long-living triplet excitons in iTMC-based LECs, they can be quenched by the n- and p-doped regions^35^. Therefore, with the growth of doped regions, the quenching of excitons increases with time and reduces the lifetime of the LEC. The dynamic p–i–n junction must be stabilized with time to maintain the high-efficiency levels achieved after applying the voltage. It was found that the lifetime of iTMC-LECs strongly improves when driven by a pulsed current mode. The pulsed current driving stabilizes the doped regions in the LEC leading to longer lifetimes^35^.

It has been shown that the turn-on time of the LEC devices is improved by increasing the conductivity of the emitting layer^36,37^. Accordingly, several studies on the addition of ionic materials to the emitter layer and chemical modification of iTMCs have been done to improve the response time of iTMC-LEC devices. For example, the addition of ionic salts (LiPF_6_) and ionic liquids to the emitter layer and attachment of ionic moiety (such as imidazolium and triethylammonium) to an ancillary ligand, leads to a decrease in the turn-on time of LECs^23,36–45^. However, by the addition of ionic species into the emissive layer, the lifetimes of iTMC-LECs reduce significantly. In a brilliant work, Tordera et al. described the iTMC-LEC device with a lifetime of over 4000 h and sub-second turn-on time under the pulsed current condition^35^. However, in some cited iTMC-LECs, one of these two items (t_1/2_ and t_on_) is not appropriate from the practical point of view (See Table S3, ESI)^38–43,46^. For example for the LECs based on [Ir(ppy)_2_(N^N)]^+^ (ppy: phenyl pyridine, N^N: aromatic diimine), Ertl and co-workers have reported a lifetime of 6000 h with a high turn-on time of 870 h under the pulsed current driving mode^33^. Another concern about iTMC-emitters is that they exhibit severe phosphorescence concentration-quenching in the solid state as a result of their relatively long triplet lifetimes which markedly suppresses the phosphorescence efficiency of the emissive layers and thereby the LEC performances. It has been shown that enclosing bulky groups (such as phenyl) to peripheral ligands can provide steric hindrance and effectively reduces self-quenching of iridium complex emitters in the solid-state form^47–50^. Phenanthroimidazole (PI) molecules with a specific structure and supreme photophysical features are frequently used as small molecules, ancillary ligands, and as a host material in optoelectronic devices. PI molecules include an imidazole moiety fused with phenanthrene or phenanthroline in which by changing the substituents at the N1 and C2 positions of the imidazole, can adjust the electronic properties of PI^51–56^. Therefore, the PI ligands with high chemical modification potential can be employed as molecularly-engineered switches to achieve efficient, stable, and fast response LECs. Chelating N, N- coordinating site containing imidazole ring (type 1) and phenanthroline moiety (type 2) are two types of strong metal-binding sites that can exist in PI ligands. It has been revealed that the LECs based on Ir-iTMC complexes with type 1 ancillary ligands show poor efficacy (lower than 0.8 cd. A^−1^)^57,58^. By contrast, the Ir-iTMC complex with type 2 PI ancillary ligand showed highly stable and efficient LEC^28^. However, to the best of our knowledge, no report has been published on the effects of chemical modification of type 2 PI ligands on the performance of Ir-iTMC-LEC devices^19^.

**Figure 1:**
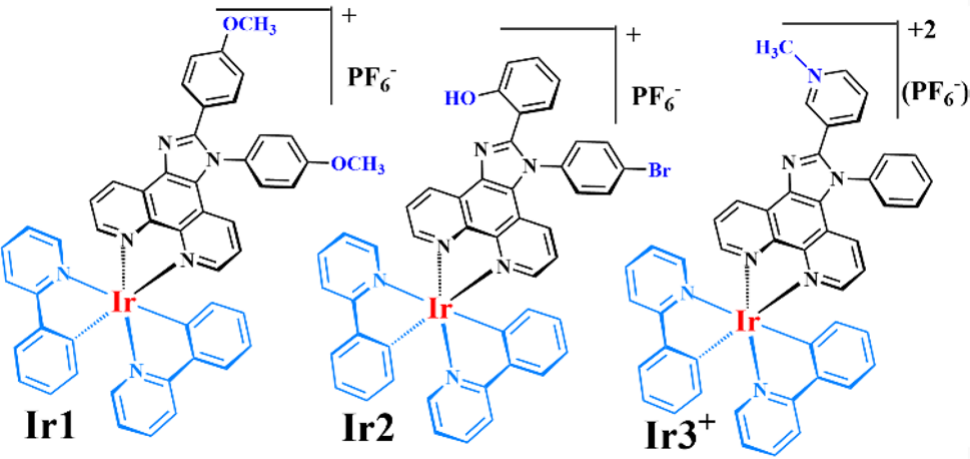
Molecular structures for emitter Ir1, Ir2, Ir3^+^.

In light of all the aforementioned facts, we designed and synthesized three novel cyclometalated iridium (III) complexes as yellow to orange emitters based on the PI ligands (L1, L2, and L3) containing an electron donor/acceptor and ionic substitutions (Fig. 1), namely, Ir1, Ir2, and Ir3^+^. In this work, a combinational approach such as bulky structure, electron donor/acceptor substitutions, and tethered ionic group are used for optimization of the device efficiency, stability, and response time of cyclometalated iridium (III) complexes-based LECs. Accordingly, the prepared solution of complexes exhibits high yellow to orange phosphorescence and reversible red/ox properties. Meanwhile, the complexes were exploited as emitters for solution-processable LECs and showed efficient and fast response electroluminescence (EL).

## Results and discussion

### Synthesis and characterization

The complete synthesis procedure of the PI ligands and their cyclometalated iridium (III) complexes are given in the electronic supplementary information (ESI) and they were fully characterized by ^1^H/^13^C NMR, elemental analysis, and TOF-mass spectrometry.

The PI ligands were synthesized and easily purified without the need for column chromatography. The prepared ligands (PI) were reacted with chloro-bridged Ir(III) dimers of [Ir(ppy)_2_Cl]_2_ in a mixture of methanol/dichloromethane to synthesize Ir1, Ir2, and Ir3. Subsequently, Ir3^+^ was obtained by reacting Ir3 with methyl iodide in acetonitrile (See Scheme 1, ESI).

### Photophysical characterizations

**Figure 2:**
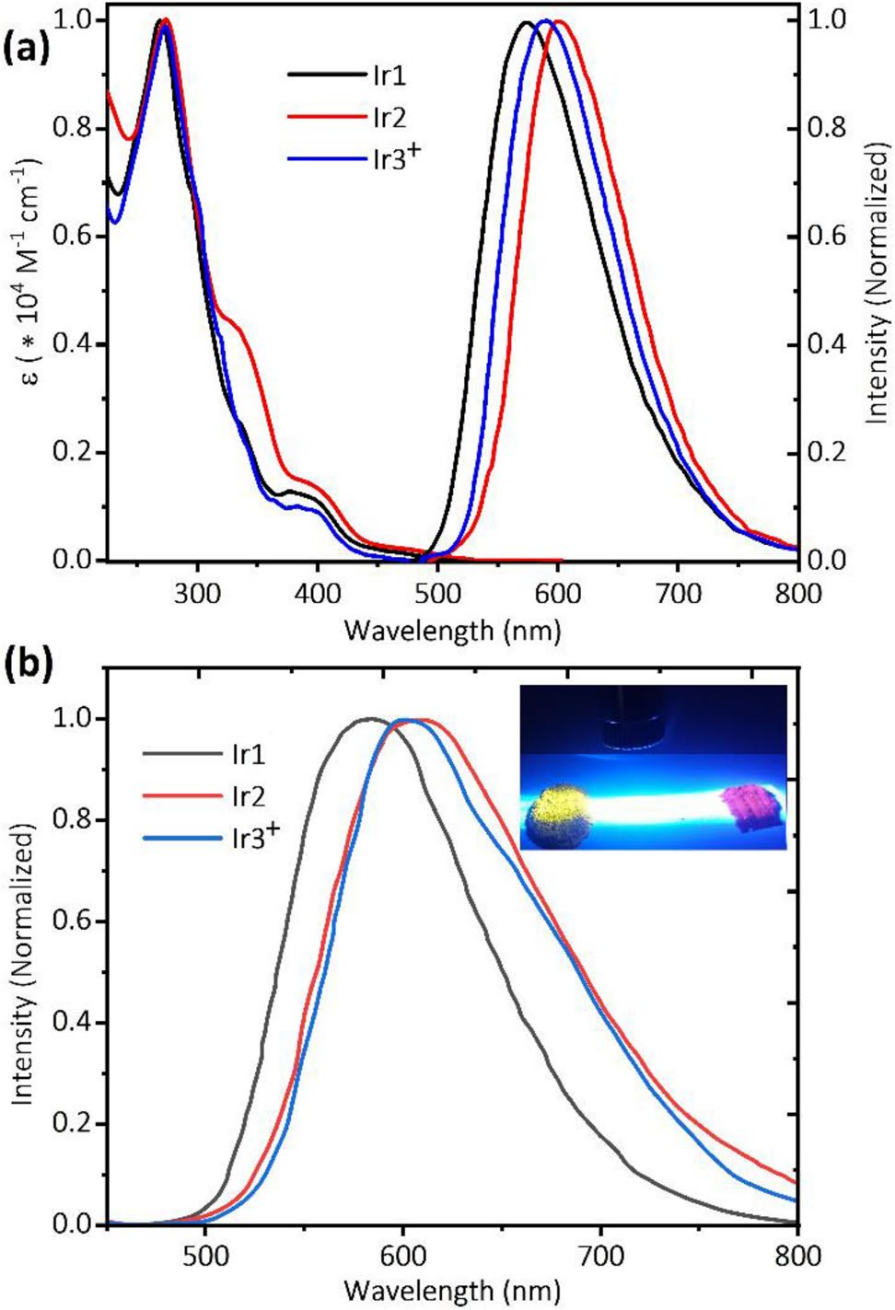
(**a**) Electronic absorption and emission spectra of iridium complexes Ir1, Ir2, Ir3^+^ in ACN solution, (**b**) PL of complexes in solid-state, inset: Ir1 (left) and Ir2 (right) powder under 405 nm irradiation.

Figure 2a depicts the room temperature UV–visible absorption (UV–Vis) and photoluminescence (PL) spectra of the complexes in acetonitrile. Detailed photophysical characteristics of these complexes and archetypal complex [Ir(ppy)_2_(phen)]^+^^59^ are given in Table 1.

**Table 1:** Photophysical data for Ir1, Ir2, and Ir3^+^.

	Absorption ${\lambda }_{abs }\left[nm\right] \left(\varepsilon /{10}^{3}\right)$^a^	PL (solution)^b^					PL (neat film)^c^		E_g Opt_^d^ (eV)
		${\lambda }_{em}$[nm]	Φ_p_ [%]	$\tau$[ns]	${k}_{r}$ $[\times {10}^{5}{s}^{-1}]$	${k}_{nr}$ $[\times {10}^{5}{s}^{-1}]$	${\lambda }_{em}$[nm]	Φ_p_ [%]	
Ir1	269 (84.2), 294 (58.3), 334 (22.1),376 (10.8), 396 (10.0), 466 (1.4)	580	46	880	5.20	6.14	584	32 (53)	2.54
Ir2	274 (82.0), 302 (50.5), 331 (35.7), 383 (12.4), 402 (11.4), 482 (1.6)	602	42	970	4.33	5.98	606	26 (38)	2.45
Ir3^+^	272 (78.0), 303 (4.89), 318 (32.8), 341 (16.0), 383 (7.8), 395 (7.5), 452 (0.6)	592	40	950	3.66	6.42	603	8 (15)	2.52
[Ir(ppy)_2_(phen)]^+^	–	583^e^	39^e^	230^e^	17.3^e^	2.7^e^	591^e^	11^e^	–

^a^In air-equilibrated CH_3_CN at 298 K (10^−5^ M).

^b^In degassed CH_3_CN at 298 K (10^–5^ M); the emission quantum yields (Φ_p_) were calculated by comparison with quinine sulfate (Φp = 0.545 in 1 M H_2_SO_4_ (estimated error of ± 5%.); Lifetime were calculated based on a mono-exponential decay model. k_r_ and k_nr_ were calculated based on the equations τ = 1/ (k_r_ + k_nr_) and Φ_p_ = k_r_/ (k_r_ + k_nr_).

^c^Solid-state absolute quantum yield was measured by employing an integrating sphere system (estimated error: ± 5%), Values in parentheses were obtained for films with a composition similar to LEC (iTMC:IL, 4:1).

^d^Optical bandgap, from the intersection of absorption and emission spectra.

^e^Data from Ref.^59^.

All the complexes showed intense absorption bands (ε > 8 × 10^4^ M^−1^ cm^−1^) in the UV region of the spectrum with a maximum in the range of 260 to 300 nm, which is attributed to the ligand-centered (LC) spin-allowed ^1^π-π* transitions involving both the cyclometallating (ppy) and ancillary (PI) ligands. The broad and less intense absorption bands between 300 and 430 nm are ascribed to spin-allowed metal-to-ligand (^1^MLCT) and ligand-to-ligand charge transfer (^1^LLCT) transitions, while the low-intensity bands beyond 430 nm correspond to the spin-forbidden ^3^MLCT, ^3^LLCT, and LC ^3^π-π* transitions of the complexes.

The spin-forbidden triplet transitions (^3^MLCT, ^3^LLCT) are partially allowed owing to the strong spin–orbit coupling of heavy iridium (III) atom to occur at a lower molar absorptivity than the corresponding singlet-allowed excitation (^1^LLCT/^1^MLCT)^60,61^. The absorption spectra in the lower-energy region for Ir2 are significantly red-shifted by 16 nm compared to those of Ir1 which is directly resulting from changing the HOMO/LUMO energy gap owning to the presence of electron-withdrawing (Br) and electron donor groups (OCH_3_) on the PI ligand of the complexes. The complexes Ir1, Ir2, and Ir3^+^ show broad structureless PL spectra corresponding to yellow-orange emission centered at 580, 592, and 602 nm, upon 350 nm photoexcitation in an argon-saturated dichloromethane solution, demonstrating the emissions in the solution arise dominantly from ^3^CT states^62^. The introduction of electron-withdrawing and electron donor groups on the aryl ring of the PI ligand exerts a negligible influence on the maximum luminescence. Complex Ir2 shows a red-shift of 19 nm in maxima emission, compared to that of the archetypal complex [Ir(ppy)_2_(phen)]PF_6_^59^. This red-shift can be explained by the electron-deficient nature of the fused imidazole moiety with Br as electron withdrawing group (L2), and π-expanded structure of the PI ligand, leading to stabilization of the LUMO and a smaller energy band gap, consequently (see optical band gap, E_g Opt_, in Table 1). For the same reason, Ir1 exhibits lower maximal emission in this series, because of the destabilization of the LUMO by two electron donor groups (OCH_3_) on the L1. Degassing the solution of Ir1, Ir2, and Ir3^+^ complexes caused high PL quantum yield (PLQY) values of 0.46, 0.42, and 0.40 and excited-state lifetimes (τ) of 880, 970, and 950 ns, respectively, which exhibit slightly higher PLQY compare to the archetypal complex [Ir(ppy)_2_(phen)]^+^ (Φ_pL_ = 0.39)^59^, which could be attributed to the rigid structure of PI ancillary ligand^63^. It is noteworthy that, the PLQY of these complexes are the highest values for yellow to orange phosphorescence among their phenanthroline-based cationic iridium complexes (See Table S3, ESI)^18,19^. Time-resolved phosphorescence decays were found to be monoexponential, indicating the presence of a single emissive species. Furthermore, radiative (k_r_) and nonradiative (k_nr_) decay rates were calculated from PLQY and τ, and are listed in Table 1. All complexes, Ir1, Ir2, and Ir3^+^ show relatively long excited-state lifetimes and smaller k_r_ values compared to the benchmark complex which suggests that their emitting triplet states should contain considerable ligand-centered ^3^π − π* character relative to the parent complex [Ir(ppy)_2_(phen)]^+^ and shielding effect of the periphery phenyl groups around the iridium core in these complexes that prevent nonradiative intermolecular charge recombination^49,64,65^.

To see the potential use of the complexes as the emitters in LEC devices, their emission properties were measured in neat films (pristine complexes without IL, see Table 1.). All complexes showed featureless emission spectra (Fig. 2b), indicating that the emission of both the solution and neat film arises from the ^3^CT state^60,61,66^. PL emission spectra of the neat films in comparison with the solutions of Ir1, Ir2, and Ir3 + are red-shifted by 4, 4, and 11 nm, respectively, that indicates the lower intermolecular interactions of PI-based complexes (Ir1, Ir2) compared to the parent complex [Ir(ppy)_2_(phen)]^+^. Nevertheless, the largest red-shift (11 nm) was observed for Ir3^+^, probably due to increased intermolecular interaction in its neat film. All complexes in neat film forms showed lower PLQY compared with their solutions (See Table 1). The major reason is associated with the close packaging of the complexes in the neat films, which promotes the self-quenching processes^49^. However, in the neat films, the PLQYs of Ir1 and Ir2 (0.32 and 0.26, respectively) are about 2.5 to threefold higher than that of [Ir(ppy)_2_(phen)]PF_6_ (0.11), even though their PLQYs in the solution are slightly different, demonstrating that the bulky phenyl groups at the PI ligands significantly suppress the phosphorescence concentration quenching^48,49^. Furthermore, by adding ionic liquid (IL) to the neat-films, the values of PLQYs significantly increased up to 0.53 and 0.38, due to decreasing the self-quenching of the emission^67^. It is noted that the Ir3^+^ complex presented a much lower PLQY in film (pristine and mixed) with respect to Ir1 and Ir2, exhibiting a higher aggregation tendency of ionic compounds in comparison with neutral ones^53^.

### Electrochemical characterizations and DFT calculations

**Figure 3:**
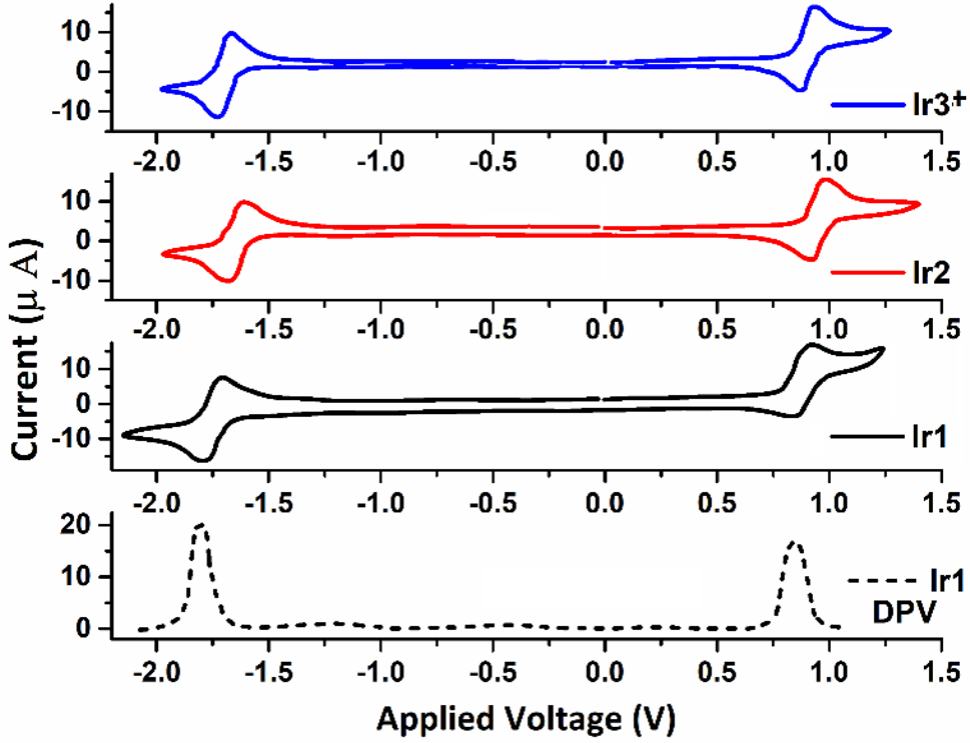
Cyclic voltammograms of Ir1, Ir2, and Ir3^+^ in degassed ACN (10^−3^ M), measured at a scan rate of 0.1 V/s and differential pulse voltammograms of Ir1 (dot line).

According to the LEC working principle, the transport of electrons and holes in metal complex-based LEC devices take place through consecutive oxidation and reduction of the metal complex during device operation. Therefore, the redox behavior of iTMCs play an important role in understanding the overall performance of LEC devices. Hence, the electrochemical properties of complexes were investigated using cyclic voltammetry and differential pulse voltammetry techniques. Figure 3 shows the cyclic voltammograms and the electrochemical data are presented in Table 2.

**Table 2:** Electrochemical properties of complexes Ir1, Ir2, Ir3^+^.

	E_1/2 ox_ (∆E)^a^ (V)	E_1/2 red_ (∆E)^b^ (V)	HOMO^c^ (eV)	LUMO^d^ (eV)	E_gap Elc._^e^ (eV)
Ir1	0.87 (81)	− 1.81 (70)	− 5.67	− 2.99	2.68
Ir2	0.91 (86)	− 1.61 (74)	− 5.71	− 3.19	2.52
Ir3^+^	0.89 (112)	− 1.70 (106)	− 5.69	− 3.10	2.59

^a^Half-wave potential, E_1/2_ = ½ (E_pa_ + E_pc_); 0.1 M acetonitrile/TBAP versus Ag/AgCl at a scan rate of 100 mV/s, Values in parentheses: difference between the anodic and cathodic peak potentials, ΔE = E_pa_ − E_pc_ (mV).

^b^The half-wave potential of reduction peak for complexes.

^c^From E_HOMO_ = − (4.8 + E_ox_) eV.

^d^From E_LUMO_ = − (4.8 + E_red_) eV.

^e^Electrochemical band gap from E_gap_ = E_HOMO_ − E_LUMO_.

As shown in Fig. 3, at the positive potential, all complexes exhibited a main reversible oxidation process, which is due to the oxidation of Ir(III) to Ir(IV) with a strong contribution from the cyclometalated ligand, ppy^68–70^. At the negative potential, all complexes possess one reversible or quasi-reversible reduction peak which is considered to be caused by phenanthroimidazoles as ancillary ligand with minor Ir(III) center involvement^28^. It is noteworthy that for most of this type of cyclometalated complex along with N^N ancillary ligand, the highest occupied molecular orbital (HOMO) has been reported to be a mixture of the d_π_(Ir) orbitals of iridium and the π orbitals of the C^N ligand while lowest unoccupied molecular orbital (LUMO) localized at N^N ancillary ligand^38^.

Therefore, to shed light on the effect of various functional groups of ancillary ligands on the electronic properties of the emitter, the HOMO and LUMO levels and electrochemical band gaps of complexes were derived from their corresponding redox potentials using empirical formulae (footnote of Table 1)^48^.

First of all, the reversible oxidation and reduction processes demonstrate the good electrochemical stability of complexes which are beneficial to achieve stable iTMC-LECs. Since, Ir3^+^ has the highest ∆E value compared to other complexes. Thus, it can be concluded that the electrochemical stability of this complex is lower than the others.

**Figure 4:**
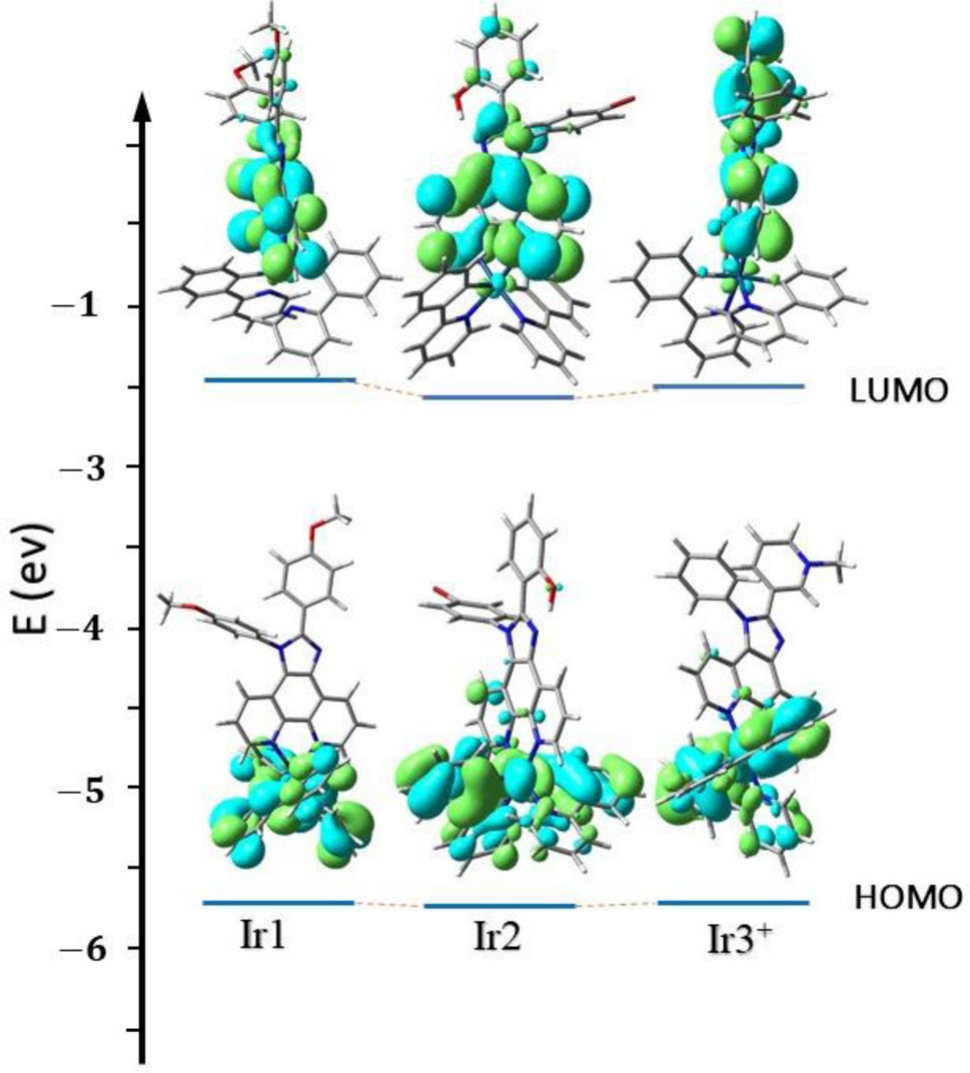
Energy levels and surface distributions of HOMO/LUMO orbitals at optimized S0 geometries computed for complexes Ir1-3^+^.

The oxidation potential of complexes is nearly identical (0.87–0.91 V) which indicates that peripheral groups on the PI ligand hardly alter the HOMO level of the complexes. However, the reduction potential of Ir1 (− 1.81 V) is significantly cathodically shifted (ca. 0.2 V) with respect to that of Ir2 (− 1.61 V), which attributes a significant destabilized LUMO for Ir2. The presence of peripheral electron-donating (OCH_3_) and withdrawing groups (Br) affect the reduction potential of the PI ligands that are destabilized and stabilized the LUMO levels of cyclometalated complexes, respectively. In the meantime, the energy gap increased for Ir1 (2.68 eV) compared with Ir2 (2.52 eV) and also led to a gradual blue-shift for the emission (22 nm). These data are further supported by DFT calculations. The computed HOMO, LUMO energy levels, and electron density contour plot (Fig. 4) reveal that: first, the substitution groups on the PI ligands have only a minor influence on the HOMO levels, but pronounced influence on the LUMO levels, which is expressed in the reduction potentials (Table S2, ESI): and second, the HOMO is mainly localized on the iridium and the cyclometalated ligand (ppy), whereas the LUMO is localized on the PI ligand (Fig. 4). These results reinforced the important role of the functionalization of the PI ligands in the electronic properties of these complexes.

### Electroluminescent properties of LECs

**Figure 5:**
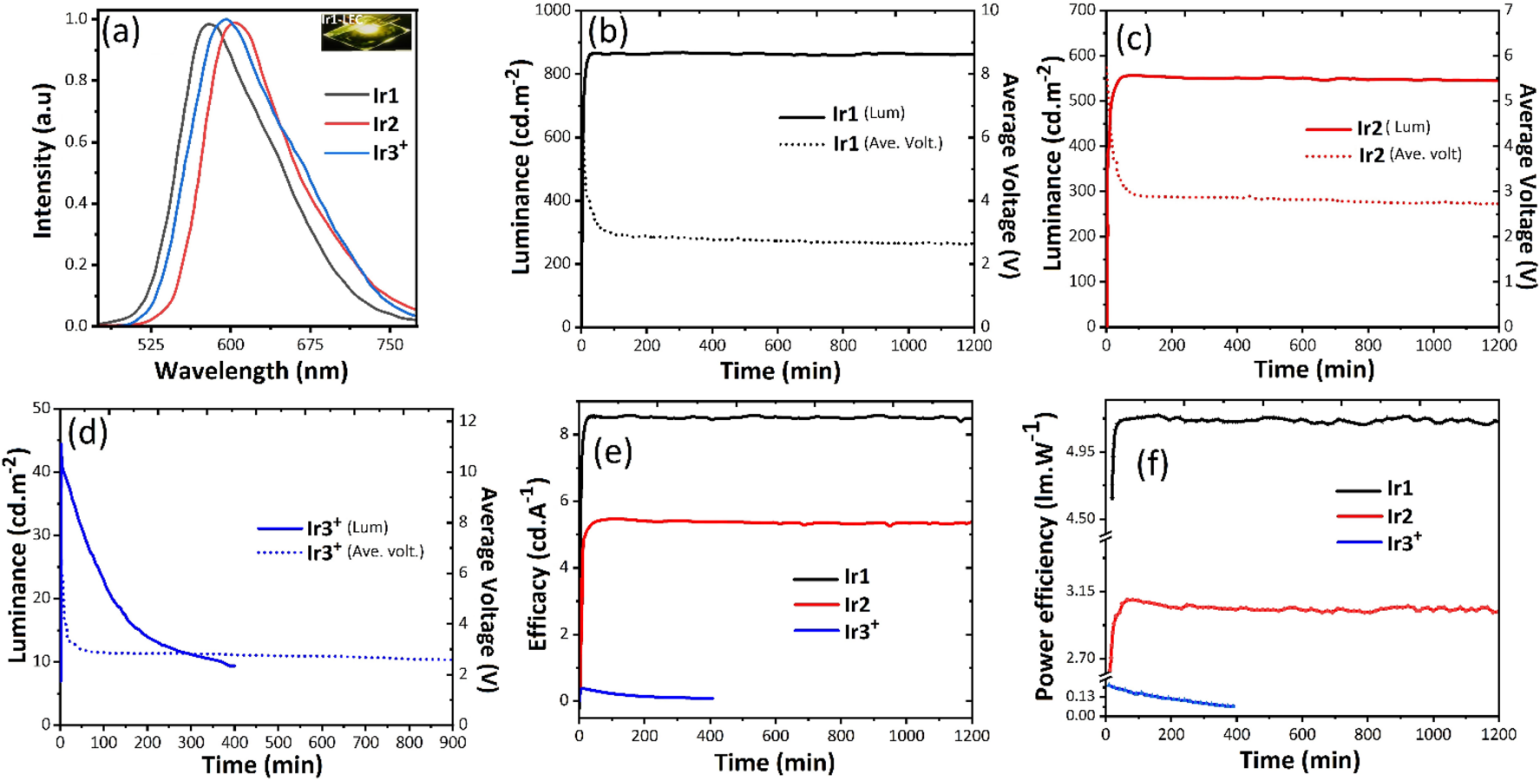
Electroluminescence spectra of LECs Ir1, Ir2 and Ir3^+^, inset: Ir1-LEC (**a**). Luminance and average voltage versus time for iTMC-LEC devices, (**b**) iTMC: Ir1, (**c**) iTMC: Ir2, (**d**) iTMC: Ir3^+^. (**e**) Efficacy vs time, (**f**) Power efficiency vs time. (All curves were obtained under pulse current driving mode at 100 A.m^−2^ and duty cycle of 50%).

To confirm the excellent phosphorescence of the complexes Ir1, Ir2, and Ir3^+^, LEC devices were fabricated on the ITO glass in a double-layer architecture consisting of PEDOT:PSS layer, to increase the reproducibility of the devices, and the emissive layer using a solution process. The active layer contained Ir-iTMC and ionic liquid (IL) 1-butyl-3-methylimidazolium hexafluorophosphate ([BMIM][PF_6_]). The incorporation of IL into the films contributed to accelerate the LEC device response (reducing the turn-on time) by increasing the concentration of ionic molecules and ionic mobility in the emitter layer. In addition, IL increases the efficiency of the devices by decreasing the concentration quenching as discussed earlier. In the end, aluminium metal (Al) was deposited on the emissive layer as cathode electrode contact. The EL data of LECs were collected at pulsed current densities 100 A.m^−2^ over time (1 kHz block wave and 50% duty cycle). More details concerning the LEC device fabrication and characterization methods can be found in the ESI. At first, considering the lifetime and maximum efficiency of the Ir1-based LEC, the optimal thickness of the emitting layer, iTMC:IL molar ratio, and average current density were obtained to be 160 nm, 4:1, and 100 A.m^−2^, respectively (See Fig. S10, ESI). Furthermore, the effect of bulky structure of Ir1 complex on the surface morphology of the spin-coated film was investigated by top-view SEM (See Fig. S9, ESI). The good solubility of the ionic complexes in the acetonitrile (specially Ir3^+^), led to pinhole-free films with smooth and good morphology quality for LEC fabrication. Figure 5a displays the EL spectra of the iTMC-LECs. EL spectra and maximum emission are closely similar to PL spectra of the complexes in thin film, meaning that the emission's nature is identical and comes from the same excited state in both excitation methods (Tables 1 and 3). The maximum EL emission of the LEC-based Ir1, Ir2, and Ir3 + were centered at 581, 605, and 596 with CIE coordinates of (0.52, 0.48), (0.64, 0.35), and (0.61, 0.39), respectively, that are correspond to yellow-to -orange emission. As already mentioned, the shorter wavelength emission of Ir1 with respect to others is due to the influence of electron-donor and electron-withdrawing groups on the PI ligand that effectively alter the LUMO levels in this series. Notably, the LECs presented a similar EL spectrum during the operation with time which is a desirable feature for LECs^18,71^ Under a block-wave pulsed current at an average current density of 100 A.m^−2^_,_ the time-dependent luminance, average voltage, efficacy, and power efficiency of LECs based on Ir1, Ir2, and Ir3^+^ were achieved. As shown in Fig. 5b–d, luminance gradually increases with time until a maximum is reached and then starts to decrease which is a typical characteristic for LEC devices^1–3,72^.

**Table 3:** Device Performance of the LEC: ITO/PEDOT:PSS/iTMC(Ir1, Ir2, Ir3^+^):[BMIM][PF_6_]/Al. Operated under a pulsed current of 100 A·m^−2^ (1000 Hz, 50% duty cycle, block wave).

Device	λ_max, EL_ (nm)	CIE [x, y]^a^	V (V)^b^	t_1/2_ (h)^c^	t_on_ (h)^d^	L_max_ (cd.cm^−2^)^e^	Efficacy (cd.A^−1^)^f^	PE_max_ (lm.W^−1^)^g^	EQE (%)^h^	EQE_Th_ (%)^i^
Ir1	581	[0.519, 0.480]	2.6	2130	0.65	870	8.60	5.16	3.1	11.8
Ir2	605	[0.648, 0.351]	2.7	1450	1.30	563	5.52	3.12	2.5	8.4
Ir3^+^	596	[0.608, 0.391]	2.6	2.25	0.15	45	0.38	0.22	0.24	3.3
[Ir(ppy)_2_(phen)]^+j^	578	–	–	73	6.4	63	–	–	2.1	–

^a^Commission Internationale de l’Eclairage color coordinates, 1931.

^b^Driving voltage (after 15 h).

^c^Lifetime: Time to reach one-half of the maximum luminance (Values obtained from extrapolation).

^d^Turn-on time: time to reach maximum luminance.

^e^Maximum luminance.

^f^Maximum efficacy: ratio luminance/average current.

^g^Maximum power efficiency.

^h^Maximum external quantum efficiency of LEC devices.

^i^Theoretical expected external quantum efficiency calculated from EQE_Th_ = bΦ_PL_/2n^2^ (b = 1, n = 1.5, Φ_PL_ = thin films PLQYs).

^j^Data from Ref.^67^.

Upon biasing the device, because of high initial injection barriers of electrons and holes, an operating voltage was observed at 5.8 V. However, at this high voltage, the ions dissociate and migrate faster towards the respective electrodes, leading to the formation of p and n-doped regions near the electrodes and the following construction of a p-i-n junction within the layer. It facilitates the electrons and holes injection from the inert electrodes, lowering the barrier for charge injection^72,73^. When the electrochemically doped layers are well-formed, the devices reach the maximum luminance values (L_max_) and the initial voltages drop to minimum stable values (2.6–2.7 V) that are nearly equal to the electrochemical band gap^74^, indicating no charge injection barrier. Additionally, the minimum average voltage of the devices remains close to the steady-state value along the device operation time, depicting that there are no signs of charge transport issues or chemical degradation in the all LECs (Fig. 5b–d)^75^. Interestingly, the initial voltage for the LECs based on Ir1 and Ir2 decreases to 3 V after about 60 min, while for Ir3^+^ this occurs after 10 min which might be related to the enhancement of ionic mobility of the emissive layer that is induced by tethered methyl pyridinium moiety (PyCH_3_^+^) on the Ir3^+^ complex^37,45^.

Under constant current of 100 A.m^−2^, the LECs based on Ir1, Ir2, and Ir3^+^ give maximum luminance of 870, 563, and 45 cd.m^−2^ and external quantum efficiency (EQE) of 3.1, 2.5, and 0.24%, respectively. These results are consistent with PLQYs recorded in the films for complexes. However, obtained EQEs are lower than the theoretical maximum efficiency (EQE_th_)^46^ (Table 3). The higher L_max_ and EQE of the Ir1-based LEC device with respect to those of others is attributed to their higher PLQY in the film. Regarding the Ir3^+^-based LEC device, it shows the lowest luminance and efficiency, revealing the high tendency of ionic iridium complex (Ir3^+^) to quench the excitons in the solid state (See PLQYs of the films in Table 1). Moreover, the L_max_ and EQE afforded by the Ir1- based-LEC device are among the highest values reported in the literature for the [Ir(ppy)_2_(N^N)]^+^ type emitter with N^N phenanthroline-based ancillary ligand (See ESI Table S3)^28,67,76–78^.

The Ir1, Ir2, and Ir3^+^ based LEC devices showed fast response with t_on_ of 0.65, 1.3, and 0.15 h, respectively. The significant improvement in response time of the Ir3^+^-based LEC device, therefore, can be attributed to the ionic nature of the emissive layer, due to the accelerated formation of the doped regions. This further proves the significant role of the ionic methyl pyridinium moiety in reducing the t_on_ of the LEC devices by up to 75–88% in this series, and the great potential of the modification of PI ligand with ionic groups.

Remarkably, the Ir1 and Ir2-based LEC device showed relatively high extrapolated half-lifetimes of 2130 and 1450 h, respectively in which the obtained t_1/2_ for the Ir1-based LEC is much higher than archetype [Ir(ppy)_2_(phen)]^+^ and their phenanthroline-based analogues. (See Table S3, ESI). Take into account, it can be ascribed to their good electrochemical stability (See section “Results and discussion”) and the presence of hydrophobic phenyl rings on their peripheral positions that limit the occurrence of water-induced substitution reaction, as well as, the use of pulsed current driving mode^21,24,25,30–34^. Therefore, the higher stability of the Ir1 based LEC device with respect to Ir2 might be attributed to the higher hydrophilicity of substitutions group on the Ir2 complex (Ph-OH, Ph-Br) respect to Ir1 (ether moiety, Ph-O-CH_3_) that it already demonstrated the effective influence of the methoxy groups on the LEC device performance^79,80^. It is worth highlighting that the lifetimes of the LECs are obtained by linear extrapolation of the time dependence of luminance and are in the same range as other very stable and efficient yellow/orange LECs which are mostly fabricated based on iridium complexes with sterically hindered N^N ligands (ranging over 1400 h)^17–19,28,81^. Compared to the parent archetype [Ir(ppy)_2_(phen)]^+^, replacing phenanthroline by a phenanthroimidazole ligand, leads to an impressive improvement in the EL properties of LEC devices with the same structure. As an illustration, t_1/2_, L_max_, and EQE of the Ir1-based LEC device compared to [Ir(ppy)_2_(phen)]^+^ increase in turn 28, 14, and 1.5 times, respectively, (See Table 2)^67^ which can be attributed to the bulky and specific structure of the PI ligand as already mentioned. Moreover, the Ir1-based LEC device demonstrates almost a ten times shorter t_on_ than the archetype-based LEC. It further indicates that complex the Ir1 is more mobile in the thin film despite of its larger size, perhaps due to the suppression of enter complex π-π stacking interactions between cation molecules.

Although, the Ir3^+^-based LEC represents a much lower t_on_ in comparison with the Ir1/Ir2-based LECs, it suffers from a relatively short half-lifetime (2.25 h). It has been shown that in general, the concentration of ionic species has a considerable effect on the lifetime and t_on_ values; the higher concentration of the ionic species leads to faster response but lower stability for LECs^42,82,83^. The different mobility of anions and cations gives rise to unbalanced charge injection/transport and movement in the recombination zone in the active layer which increases the quenching of the excitons in the recombination zones (off-centered recombination zones) and thus deteriorates the device efficiency and lifetime^37,84^. However, further modification such as the selection of bulky counter anion (for example tetraphenylborate, ph_4_B^−^, Ref.^85^) and use of various ionic additives into emissive layer^36,86^ will contribute to creating the LECs based on extra ionic complexes with acceptable stability and performance.

Furthermore, the current and power efficiency (Fig. 5e,f) and luminance versus time plots also follow similar trends that emphasize the high stability of the yellow LEC. Under the same conditions, the maximum luminance (L_max_) of all LEC devices becomes higher when the average current density is increased from 100 A.m^-2^ to 200 A.m^-2^ (Fig. S11, ESI). This achievement, however, comes at a price of lower extrapolated lifetime and efficiency for each Ir1, Ir2, and Ir3^+^-based LEC (Table S1, ESI).

Overall, this work clearly states that a rationalized choice of the various substitution on the PI ancillary ligands can deeply affect not only the emission color but also the device performance, and confirming the advantages of employing the phenanthroimidazole as an ancillary ligand for modification of the Ir(III) metal-based emitter toward the achievement of efficient, stable and fast response LECs.

## Conclusions

In conclusion, three novels Ir(III) complexes Ir1, Ir2, and Ir3^+^ were designed and successfully synthesized based on phenyl pyridine and phenanthroimidazole (PI) as cyclometalated and ancillary ligand, respectively, in which PI was functionalized with various functional groups. The complexes exhibit yellow-to-orange emission with PLQYs of up to 38% in both solution and mixed thin film, as well as good electrochemical stability. Meanwhile, the experimental data were corroborated with a computational study of the complexes that revealed the significant effect of the ligand functionalization with electron-donor and electron-withdrawing groups on the electronic properties of complexes, leading the emission ranging from yellow to orange hue. Moreover, the fabricated yellow-to-orange LEC devices by these new bulky phosphorescent complexes, accomplish half-lifetime over 2100 h, EQE over 3%, luminance exceeding 800 cd.m^−2^, and improvement of the device turn-on time by 75–88%. Eventually, the incorporation of phenanthroimidazole with various substitution groups as an N^N ancillary ligand was confirmed as an efficient and easy strategy to obtain iTMC-based LECs with long half-lifetimes, short turn-on times, and high luminance opening new door(s) in the opto-electronic application.

## Supplementary Information

Supplementary Information.

## Data Availability

All data generated or analysed during this study are included in this published article and its supplementary information file.
