# Supplementary material for: Phenanthroimidazole as molecularly engineered switch for efficient and highly long-lived light-emitting electrochemical cell
Source: Sci Rep. 2023 Feb 9;13:2287. doi: 10.1038/s41598-023-29527-7 (PMC9911647; doi:10.1038/s41598-023-29527-7)
Supplement: Supplementary file 1 — Supplementary Information. [file 41598_2023_29527_MOESM1_ESM.docx]

**Supplemenatry Information for**

Phenanthroimidazole as Molecularly Engineered Switch for Efficient and Highly Long-lived Light-Emitting Electrochemical Cell

Babak Nemati Bideh ^a^*, Majid Moghadam^b^*, Ahmad Sousaraei ^c, d^, Behnoosh shahpoori Arani ^b^

^a^ Inorganic Chemistry Department, Faculty of Chemistry, Bu-Ali Sina University, Hamedan, Iran.

^b^ Inorganic Chemistry Department, Faculty of Chemistry, University of Isfahan, Isfahan, Iran.

^c^ Institut des Matériaux Poreux de Paris, Ecole Normale Superieure, PSL University 75005 Paris, France.

^d^ Departamento de Quimica Inorganica Facultad de Ciencias, Universidad Autonoma de Madrid, Spain.

Contents of supplementary Information

[1. Methods and Materials S2](#_Toc122594466)

[2. Synthesis and characterization S3](#_Toc122594467)

[3. NMR spectra S5](#_Toc122594468)

[4. Mass spectra S8](#_Toc122594469)

[5. SEM figures S9](#_Toc122594470)

[6.Electroluminescence S9](#_Toc122594471)

[7. Tables S10](#_Toc122594472)

[References: S15](#_Toc122594473)

# 1. Methods and Materials

All reagents and solvents were purchased from commercial sources and used without further purification.NMR spectra were recorded on a Bruker 250 MHz spectrometer with CDCl_3_, D_6_-DMSO and tetramethylsilane (TMS) as solvent and internal reference, respectively. Elemental analyses were performed on Elementar Vario EL CHN elemental analyzer. TOF-Mass were performed on LC-MS/MS "Quattro Micro API micromass Waters 2695. Scanning electron microscope (SEM) was perform by TESCAN Vega Model. IR spectra were recorded on a Perkin-Elmer 597 spectrometer.The Electrochemical studies of Iridium(III) complexes (2×10^−3^M) were performed under a dry N_2_ atmosphere at 298 K by using SAMA500 potentiostat electrochemical analyzer with conventional three electrode cell, a Pt disk as the working electrode, a Pt wire as the counter electrode, and Ag/AgCl as the reference electrode. The CV measurements were performed at room temperature using 0.10 M tetrabutylammonium perchlorate (TBAP) as the supporting electrolyte and degassed acetonitrile as the solvent. In CV the following parameters and relation were used: scan rate, 100 mV s^-1^; formal potential E^ο′^ = (E_pa_ +/E_pc_)/2 where E_pa_ and E_pc_ are anodic and cathodic peak potentials, respectively; ∆E_p_ is the peak-to-peak separation. The oxidation (E_ox_) was used to calculate the HOMO/LUMO energy levels and electrochemical energy gap energy (E_gap Elc_) using the equations ^c^ From E_HOMO_ = -(4.8 + E_ox_) eV. ^d^ From E_LUMO_ = -(4.8 + E_red_) eV. ^e^ Electrochemical band gap from E_gap_ = E_HOMO_ - E_LUMO_, which is the half-wave oxidation potential of ferrocene was found to be 0.43 V [1,2]. The optical energy gap (E_g Opt_) was calculated from the intersection of absorption and emission spectra in acetonitrile solution. UV–visible absorption spectra was recorded on an Ultrospec3100 pro spectrophotometer in acetonitrile solutions at 298 K. Photoluminescence (PL) emission spectra of ruthenium complexes in degassed solutions at 298 K was recorded using Varian-Cary Eclipse flourocence spectrophotometer. The PL and PLQYs in neat films were measured with an absolute PLQY measurement system (Hamamatsu C11347) equipped with an integrating sphere. The excited-state lifetime measurements were carried out on a HydraHarp Picoquant multichannel time correlator. Excitation was provided using a 405 Sepia picosecond diode laser delivering. pulses of less than 130 ps with 2 MHz repetition rate. Single wavelength detection was conducted with a thermo-electrically cooled Hamamatsu photomultiplier coupled to a 0.5mSP-2558 Princeton Instruments (Acton Research) spectrometer equipped with a 600 lines/mm grating. PL quantum yields (PLQYs) were measured in acetonitrile solutions at an excitation wavelength of 420 nm with quinine sulfate (Φp = 0.545 in 1 M H_2_SO_4_) as the reference substance using to the well-known following equation (Eq. S2):

$$\emptyset_{unk}=\emptyset_{std}.(\frac{I_{unk}/A_{unk}}{A_{std}/I_{std}}).{(\frac{\eta_{unk}}{\eta_{std}})}^{2}$$

In equation, Φ_unk_ is PL quantum yield of ruthenium complexes, I_unk_ and I_std_ are the integrated areas of the corrected PL spectra of the ruthenium complexes and standard respectively, A_unk_ and A_std_ are the absorbances of the ruthenium complexes and the standard at the excitation wavelength (λ_exc_= 450 nm), and η_unk_ and η_std_ are the indexes of refraction of the respective solvents (taken to be equal to the neat solvents in both cases). Neat films of cationic Iridium complexes (thickness of about 160 nm) for study of solid emission were obtained by spin-coating of spectrophotometric grade acetonitrile solution of complexes (5% (w/v)) on a quartz substrate at 1000 rpm for 25 s. After evaporation of the solvent in glove-box, the neat films were dried overnight under vacuum at room temperature. The theoretical external quantum efficiency (EQE_Th_) is defined as:

${EQE}_{Th}=\frac{b\varphi}{{2n}^{2}}$

where b is the recombination efficiency (equal to 1 for two ohmic contacts), $\varphi$ is the fraction of excitons that decay radiatively, and n is the refractive index of the glass substrate and is equal to 1.5 (the factor 1/2n^2^ accounts for the coupling of light out of the device). As Ir(III)-based complexes can efficiently harvest both singlet and triplet excitons, $\varphi$ should resemble the photoluminescence (PL) efficiency. Hence, the efficiency of the device is mainly determined by the PL quantum efficiency (PLQE) of the iTMC emitter in a solid film [3].

**Device fabrication and measurement :** Indium tin oxide (ITO) coated glass with a sheet resistance of 15 Ω/square and size of 1.5 × 2.0 cm was used as the transparent anode. After being sufficiently cleaned in an ultrasonic bath containing mixed solvents such as ethanol, acetone, and isopropyl alcohol for 30 min, it was dried in the oven at 110°C for 2h followed by UV-ozone treatment for 20 min. Thin films (ca. 35 nm) of poly(3,4-ethylenedioxythiophene):poly(styrene sulfonate) (PEDOT:PSS) were spin-coated onto ITO/glass substrates at 2000 rpm for 20 s and then baked at 120 °C for 30 min in ambient air. The emissive layers (160 nm) containing Ir(III) complexes and ionic liquid (IL) 1-butyl-3-methylimidazolium hexafluorophosphate ([BMIM][PF_6_]) at a 4:1 (iTMC:IL) molar ratio were spin-coated from a 5% (w/v) acetonitrile solution at 1000 rpm for 25 s . All solution and film preparation were performed under ambient conditions. After spin coating, the devices have been transferred to an inert atmosphere glove box (<0.1 ppm O_2_ and H_2_O) and heated in 70 ^o^C for 8h to completely removed the solvent. Then, the Al electrode (70 nm) was deposited and encapsulated into the glove-box. The thicknesses of the films have been measured with an Ambios XP-1 profilometer. The active area of the devices were 0.16 cm^2^. All EL measurement were carried out in air atmosphere. The current density, luminescence versus the voltage and emission characteristics of LEC devices were measured using an AvaSpec-125 Fiber Optic spectrophotometer, a SAMA500 electroanalayzer system and a True Color Sensor MAZeT (MTCSiCT Sensor) with a Botest OLT OLED Lifetime-Test System.

**DFT calculation:** The DFT calculations were caried out using the Gaussian 03 suite of programs. The geometries of the complexes were optimized by using the B3LYP functional, and the Ir atom was treated by the LANL2DZ basis set, while all other atoms were treated by the 6-31G(d,p) basis set.

# 2. Synthesis and characterization

The compounds [Ir(ppy)_2_Cl]_2_ and 1,10-phenanthroline-5,6-dione (phendione) were synthesized according to reference methods [4, 5].

**General procedure for synthesis of phenanthroimidazole Ligands:**

Aromatic aldehyde (1 mmol) and aniline derivative (1 mmol) were dissolved in glacial acetic acid (8 mL, deoxygenated by bubbling with N_2_) and stirred for 30 min at room temperature to give a yellow solution. 1,10 phenanthroline-5,6-dione (1mmol) and ammonium acetate (770 mg, 10 mmol, excess) were then added. The reaction mixture was stirred and refluxed at 125 °C under a nitrogen atmosphere for duration of 24 hrs. After this time, it was cooled to room temperature and diluted with 30 mL cool water. The suspension mixture neutralized with aqueous ammonia solution (25%). The organic compound was extracted with 50 mL of CH_2_Cl_2_ and then removed the solvent by rotary evaporation and the residue was washed with cool ethanol and acetone. The solid was dissolved in a minimum of CH_2_Cl_2_ and then added 5-to-8-fold acetone. The clear solution keeps in refrigerator at -5 ^o^C overnight and then the precipitate was isolated. This procedure was repeated one more time to give the product as a white powder.

**1,2-bis(4-methoxyphenyl)-1H-imidazo[4,5-f][1,10]phenanthroline (L1):** Yield: 59%. mp. 287°C, Anal. calcd. for C_27_H_20_N_4_O_2_: C, 74.98; H, 4.66; N, 12.95. Found: C, 74.97; H, 4.67; N,12.93. IR (KBr): $\tilde{\nu}=$ 3025 (C-H aromatic), 1625 (C=C), 1581 (C=N), 1384 cm^-1^. ^1^HNMR (250 MHz, CDCl3): 9.17-9.10 (m, 2H), 9.26 (d, 1H), 7.7 (m, 1H), 7.61-7.45(m, 3H), 7.46 (d, 2H), 7.31 (m, 1H), 7.12 (d, 2H), 6.84 (d, 2H), 3.95 (s, 3H) (hydrogen of methoxy), 3.80 (s, 3H) (hydrogen of methoxy). ^13^CNMR (62 MHz, CDCl_3_): 160. 49, 160.25, 152.36, 148.74, 147.60, 144.64, 144.23, 135.80, 130.62, 130.49, 130.36, 129.79, 127.82, 126.82, 123.90, 123.34, 122.32, 122.03, 119.87, 115. 48, 113.78, 55.62 (Carbon of methoxy group), 55.22(Carbon of methoxy group).

**2-(2-Hydroxyphenyl)-1-(4-Bromophenyl)-1H-imidazo[4,5-f][1,10]phenanthroline (L2):** Yield: 64%. mp. 293°C, Anal. calcd. for C_25_H_15_BrN_4_O: C, 64.253; H, 3.246; N, 11.993.Found: C, 64.249; H, 3.240; N, 11.997.IR (KBr): $\tilde{\nu}=$ 3439 (O–H), 3030 (C-H aromatic), 1623 (C=C), 1586 (C=N), 1384 cm^-1^.^1^HNMR (250 MHz, CDCl3): 13.17 (s, 1H), 9.21(t, 1H), 9.09(t, 1H), 8.98 (t, 1H), 7.91 (d, 2H), 7.77 (dd, 1H), 7.55 (d, 2H), 7.36 (d, 2H),7.27 (d, 1H),7.15 (d, 1H),6.78 (d, 1H),6.62 (t, 1H).

**1-phenyl-2-(pyridin-3-yl)-1H-imidazo[4,5-f][1,10]phenanthroline (L3) :** Yield: 49%. mp. 285°C, Anal. calcd. For C_24_H_15_N_5_ (%): C, 77.20; H, 4.05; N, 18.76. Found (%):C, 77.21; H, 4.07; N, 18.77. ^1^HNMR (250 MHz, CDCl_3_): 9.16 (d, 1H), 9.05 (m, 2H), 8.77 (s, 1H), 8.55 (d, 1H), 7.88 (d, 1H), 7.78-7.60 (m, 3H), 7.50 (m, 3H), 7.39 (m, 1H), 7.26 (d, 2H) ^13^CNMR (62 MHz, D_6_-DMSO): 149.89, 149.67, 149.17, 148.16, 144.47, 137.44, 136.33,130.78, 130.44, 128.62, 127.97, 126.24, 123.58, 123.1, 122.17, 119.63.

General procedure for synthesis of complexes: [Ir(ppy)_2_(L1, L2, L3)](PF_6_)

0.0935 mmol of [(ppy)_2_Ir(µ-Cl)]_2_ (100 mg) and 0.187 mmol of phenanthroimidazole ligand (L1-3) was added to a mixture of dichloromethane and methanol (20/2.5 ml) and deoxygenated by bubbling with N_2_ and heated under N_2_ atmosphere at 42 °C for 24.0 h to give a clear orange solution. It was evaporated to dryness. The residue was purified by column
chromatography on silica with dichloromethane-methanol (100/4, v/v) as an eluent. The mainly yellow band was collected. The solvent was removed under reduced pressure and yellow solid were obtained. The solid was dissolved in 2 to 3 ml methanol and added drop-wise to a 10 ml saturated stirred aqueous solution of NH_4_PF_6_. The suspension was filtered and washed with water and ether and dried under vacuum to obtained yellow solid.

**[Ir(ppy)_2_(L1)](PF_6_) (Ir1).** Yield: 63%. IR (KBr): $\tilde{\nu}=$ 3085 (C-H aromatic), 2930 (C-H aliphatic), 1621 (C=C), 1593 (C=N), 837 (PF_6_) cm^-1^. ^1^HNMR (400 MHz, D_6_-DMSO): 9.42 (d,1H), 8.35 (d, 1H), 8.27 (dd, 1H), 8.10–7.92 (m, 3H), 7.89–7.67 (m, 4H), 7.60–7.38 (m, 8H), 7.3-7.23 (m, 2H), 7.19–7.10 (m, 2H), 7.08–6.95 (m, 2H), 6.92–6.86 (m, 4H), 6.45–6.39 (m, 2H), 3.91 (s, 3H, OCH_3_), 3.875 (s, 3H, OCH_3_). ^13^CNMR (100 MHz, D_6_-DMSO): 167.16, 166.9, 159. 41, 159.15, 151.36, 151.21, 149.6, 149.1, 148.71, 147.60,147.5, 144.23, 144.45, 144.13, 138.51,138.32, 135.60, 131.7, 130.91, 130.92, 129.77, 129.4, 127.52, 126.62, 125.11, 124.83, 123.93, 123.73, 123.62, 122.09, 122.00, 119.85, 119.7, 119.52, 114. 38, 113.61, 55.58 (Cabon of methoxy group), 55.12(Cabon of methoxy group).Anal. calcd. For C49H38F6IrN6O3P.H2O (%): C, 53.70; H, 3.49; N, 7.67. Found (%): C, 53.67; H, 3.48; N, 7.68. TOF-MS (CH_3_OH, m/z): 933.24 ([Ir1-PF_6_]^+^) (Calc. 933.25).

**[Ir(ppy)_2_(L2)](PF_6_) (Ir2).** Yield: 69%. IR (KBr): $\tilde{\nu}=$ 3069 (C-H aromatic), 1618 (C=C), 1608 (C=N), 832 (PF_6_) cm^-1^. ^1^HNMR (400 MHz, D_6_-DMSO): 11.25 (s, 1H, Hydroxy group), 9.31 (d,1H), 8.42 (d, 1H), 8.28- 8.18(m, 2H), 8.12–7.96 (m, 2H), 7.85–7.69 (m, 5H), 7.56–7.35 (m, 7H), 7.31-7.12 (m, 4H), 7.00–6.90 (m, 2H), 6.90–6.71 (m, 4H), 6.51–6.40 (m, 2H). ^13^CNMR (100 MHz, D_6_-DMSO): 168.30, 167.85, 161.20, 154.45, 150.11, 149.34, 148.53, 148.41, 147.66, 144.95, 144.38, 143.60, 141.85, 137.20, 136.68, 133.12, 132.54, 131.77, 130.68, 129.68, 129.19, 128.43, 128.02, 127.90, 126.70, 125.52, 124.85, 124.73, 123.26, 123.22, 122.81, 122.73, 120.08, 111.31. Anal. calcd. For C_47_H_31_BrF_6_IrN_6_OP (%): C, 50.72; H, 2.81; N, 7.55. Found (%): C, 50.67; H, 2.78; N, 7.53. TOF-MS (CH_3_OH, m/z): 967.21 ([Ir2-PF_6_]^+^)(Calc. 967.14).

**[Ir(ppy)_2_(L3-CH_3_)](PF_6_)_2_ (Ir3^+^).** The precursor complex Ir3 was synthesized according to the general procedure as mentioned above.Then 0.1 mmol of Ir3 (102 mg) in 5 ml acetonitrile was degassed and 0.5 mmol of methyl iodide was added to the solution and heated under N_2_ at 35 °C for 8 h. After, the solvent was removed under reduced pressure, the residue was dissolved in 5 ml methanol and precipitated by dropwise addition of saturated NH_4_PF_6_ solution. The product was collected by centrifuge and several times washed with deionized water and purified by column chromatography on silica with dichloromethane-methanol (100/4, v/v) as an eluent. Yield: 46%. ^1^HNMR (400 MHz, D_6_-DMSO): 9.21 (d,1H), 9.03 (s, 1H), 8.61 (d, 1H), 8.39-8.22 (d, 2H), 8.03 (m, 1H), 7.79–7.61 (m, 6H), 7.60–7.19 (m, 10H), 7.06–7.16 (m, 3H), 7.00–6.93 (m, 2H), 6.86–6.71 (m, 2H), 6.53–6.41 (m, 2H), 4.41 (s, 3H, Hydrogens of methyl pyridinium moiety of PI). ^13^CNMR (100 MHz, D_6_-DMSO): 167.03, 166.90, 151.7, 150.02, 149.75, 149.67, 149.37, 149.21, 148.76, 147.5, 146.23, 144.57, 144.4, 138.5, 137.34, 136.39, 131.81, 130.91, 130.75, 130.14, 129.5, 128.72, 126.97, 126.63, 125.15, 124.78, 123.58, 123.44, 123.16, 122.63, 122.17, 120.03, 119.63. 119.52. Anal. calcd. For C_47_H_34_F_12_IrN_7_P_2_ (%): C, 47.88; H, 2.91; N, 8.32, Found (%): C, 47.51; H, 2.88; N, 8.26. TOF-MS (CH_3_OH, m/z): 1034.11 (Calc. 1034.21) ([Ir3^+^-PF_6_]^+^), 444.38 (Calc. 444.60) ([Ir3^+^-2PF_6_]^2+^).


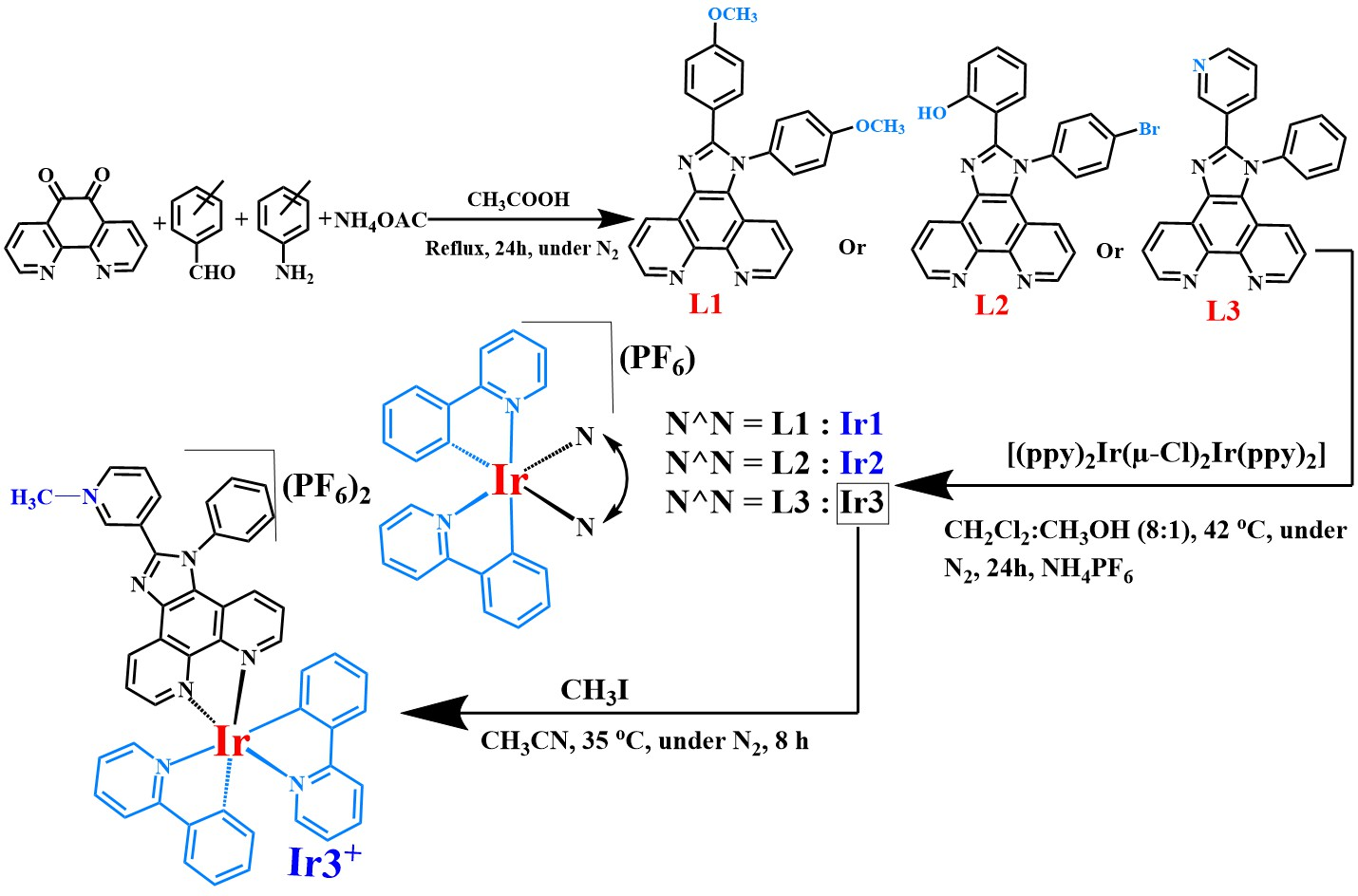


Scheme S1. Synthesis of derivatives phenanthroimidazole ligand and their cyclometalated iridium (III) complexes (Ir1, Ir2 and Ir3^+^).

# 3. NMR spectra


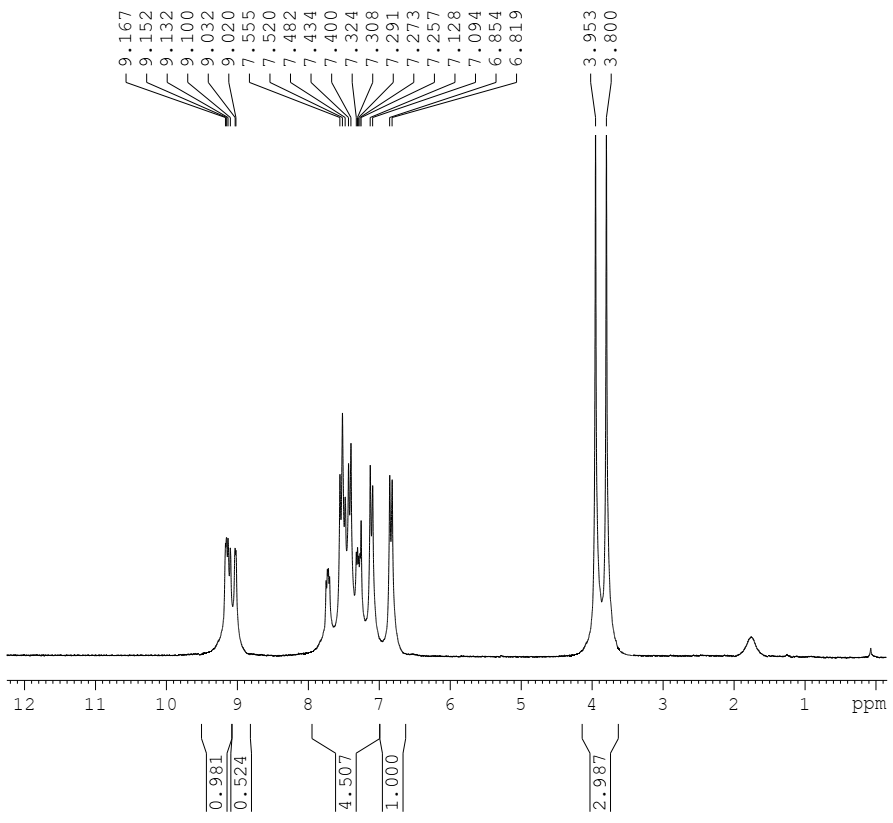


**Figure S1**. ^1^HNMR of L1 in CDCl_3_


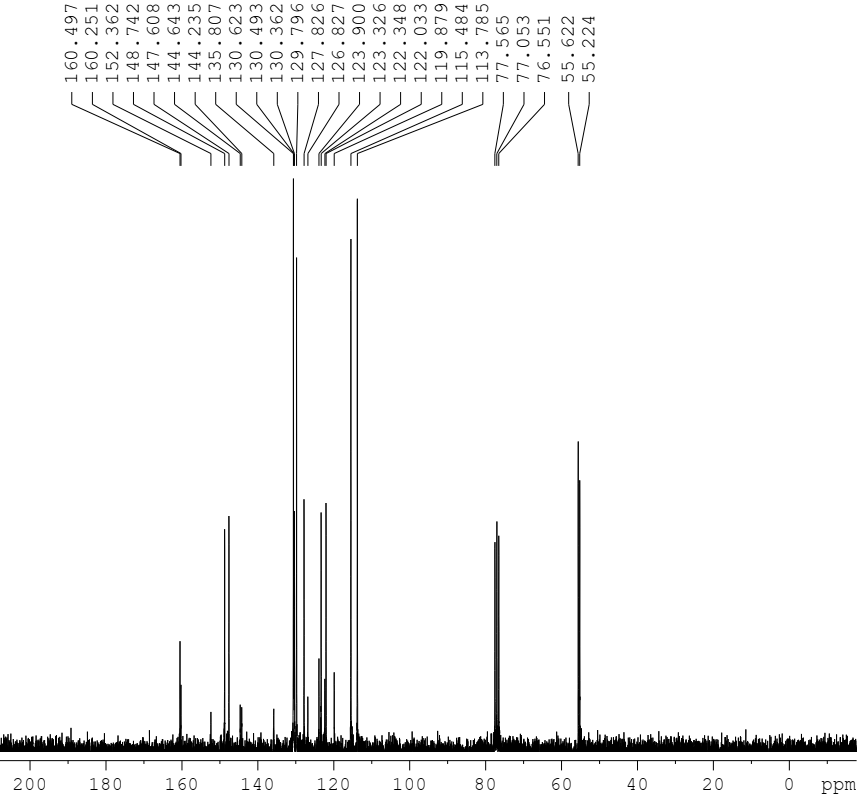


**Figure S2**. ^13^CNMR of L1 in CDCl_3_


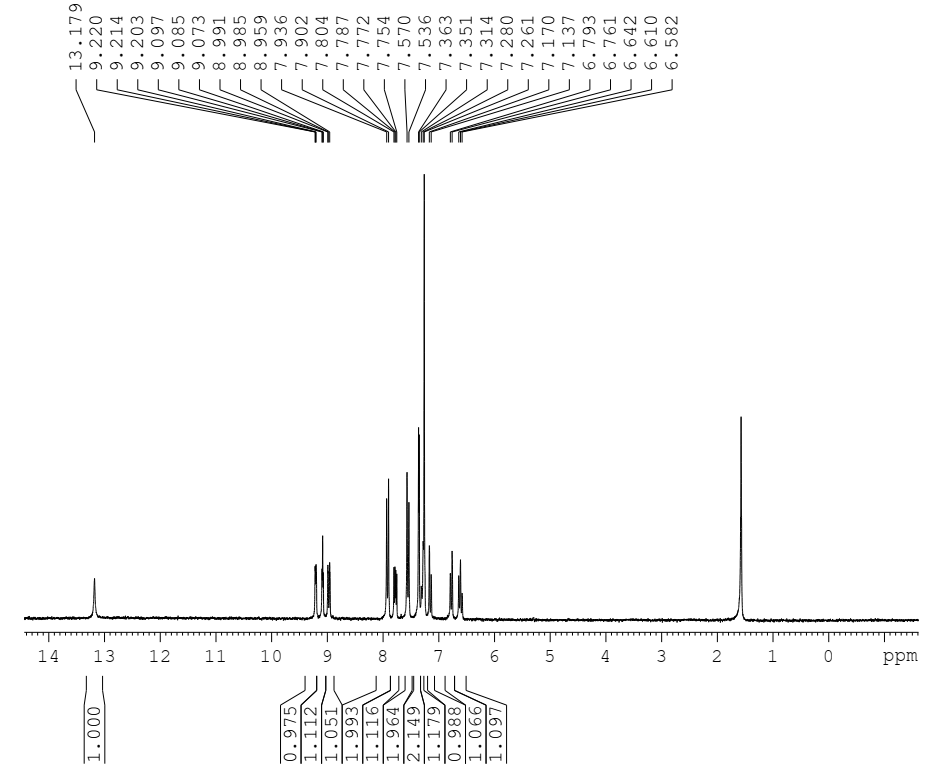


**Figure S3**. ^1^HNMR of L2 in CDCl_3_


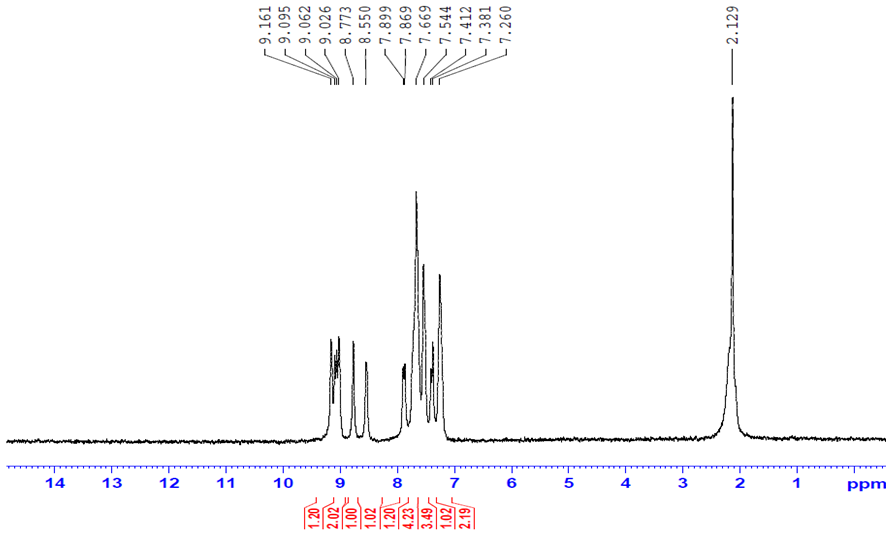


**Figure S4**. ^1^HNMR of L3 in CDCl_3_


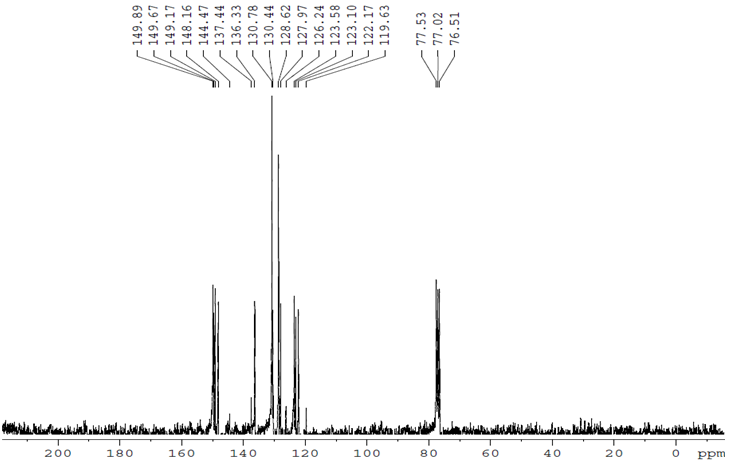


**Figure S5**. ^13^CNMR of L3 in CDCl_3_

# 4. Mass spectra


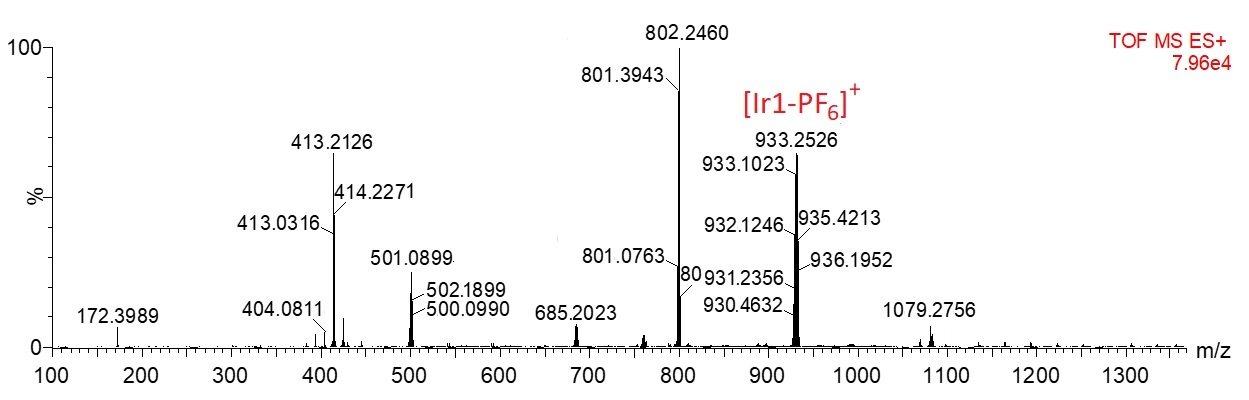


**Figure S6.** TOF-MS spectrum of Ir1


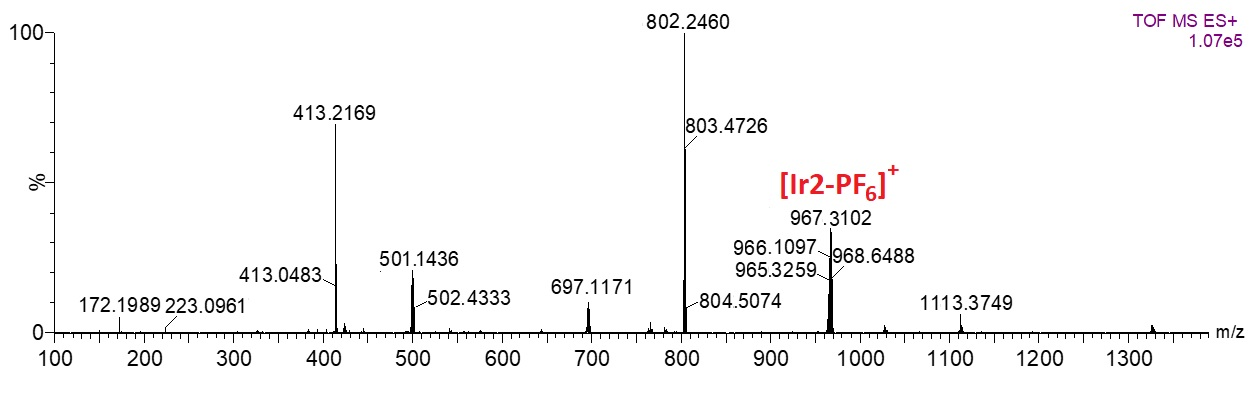


**Figure S7.** TOF-MS spectrum of Ir2


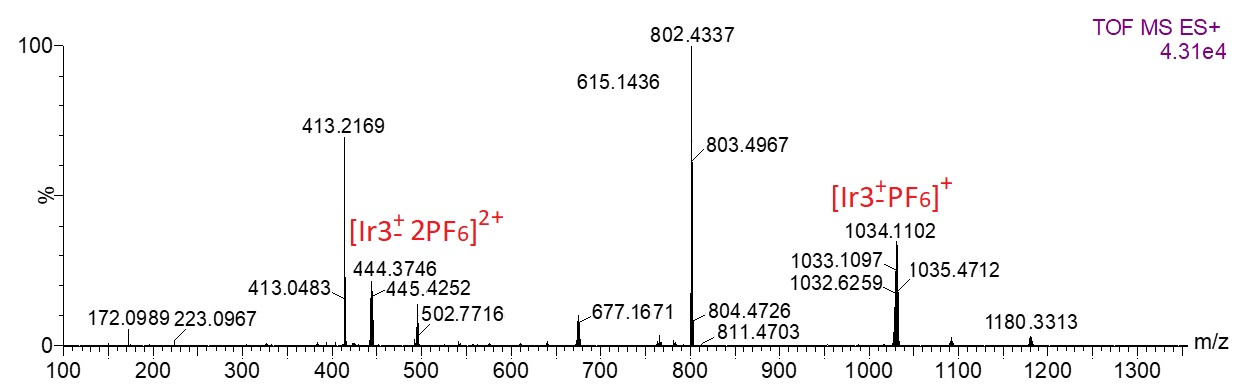


**Figure S8.** TOF-MS spectrum of Ir3^+^

# 5. SEM figure


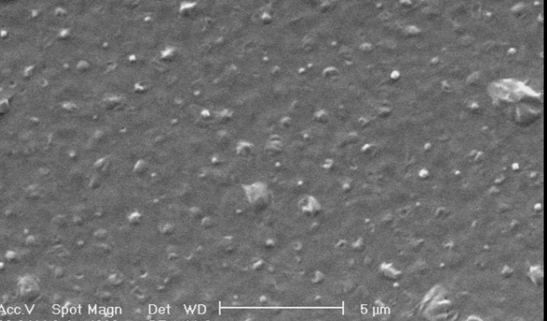


**Figure S9.** Top view SEM images of surfaces of ITO coated with Ir1 complexes.

# 6.Electroluminescence


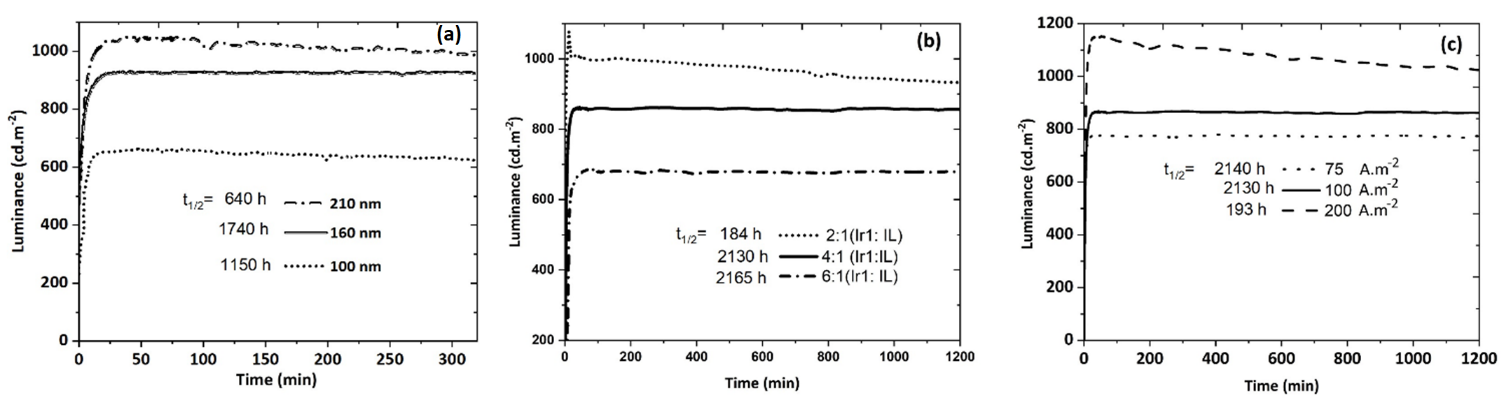


**Figure S10.** Time dependent luminance of Ir1-LEC devices with different: emitter layer thickness at 4:1 (Ir1:IL) and current density of 150 A.m^-2^ (a), Ir1: IL ratio at 100 A.m^-2^ and thickness of about 160 nm (b), current density with duty cycle of 50% (c).


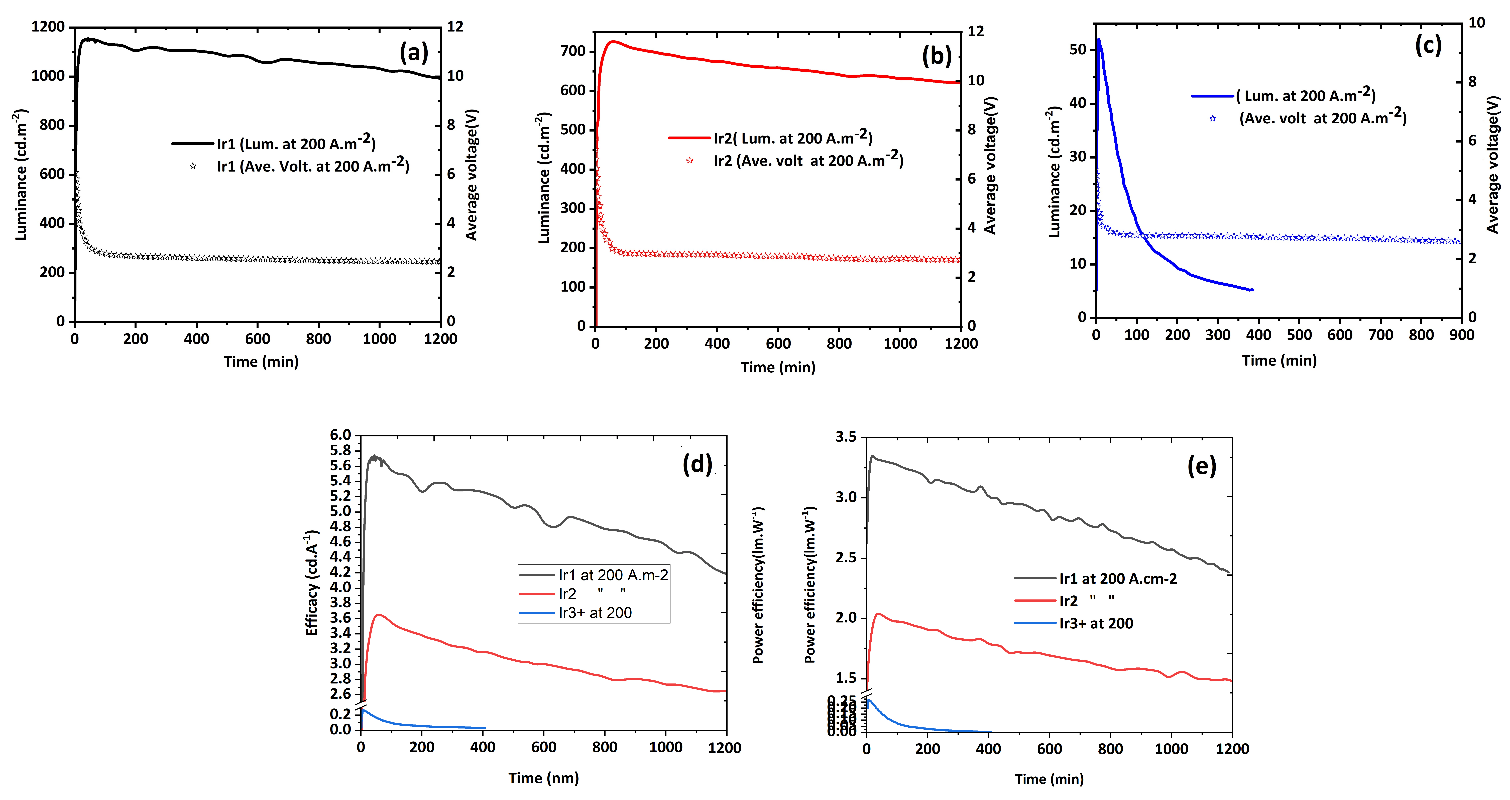


**Figure S11.** Time dependent of luminance (a, b, c), efficacy (d) and power efficiency (e) for Ir1, Ir2, Ir3^+^ based-LEC devices under pulse current density of 200 A.m^-2^ with 50% duty cycle and ratio of 4:1 (iTMC:IL)

# 7. Tables

**Table S1.** Device Performance of the LEC: ITO/PEDOT:PSS/iTMC(Ir1, Ir2, Ir3^+^):[BMIM][PF_6_]/Al. Operated under a Pulsed Current of
200 A·m^-2^ (1000 Hz, 50% Duty Cycle, Block Wave).

| LEC device | λ_max, EL_ (nm) | t_1/2_ (h)^a^ | t_on_ (h) ^b^ | L_max_  )cd. cm^−2^( ^c^ | efficacy (cd. A^-1^) ^d^ | PE_max_ (lm. W^-1^) ^e^ |
| --- | --- | --- | --- | --- | --- | --- |
| Ir1 | 582 | 182.5 | 0.58 | 1160 | 5.75 | 3.38 |
| Ir2 | 604 | 152.2 | 1.10 | 728 | 3.62 | 2.08 |
| Ir3^+^ | 593 | 1.10 | 0.10 | 53 | 0.28 | 0.24 |
| ^a^Lifetime: Time to reach one-half of the maximum luminance (Values obtained from extrapolation). ^b^ Turn-on time: time to reach maximum luminance. ^c^ Maximum luminance. ^d^Maximum efficacy: ratio luminance/average current. ^e^ Maximum power efficiency. | | | | | | |

**Table S2.** The energy levels of Ir1, Ir2. and Ir3+ complexes calculated using B3LYP/(6-31G(d,p)+LANL2DZ) in the solution phase

(acetonitrile) based on the optimized S_0_ geometries.

| Comp. | HOMO energy (eV) | LUMO energy (eV) | Band gap (eV) |
| --- | --- | --- | --- |
| Ir1 | -5.71 | -2.47 | 3.24 |
| Ir2 | -5.72 | -2.53 | 3.19 |
| Ir3 | -5.71 | -2.50 | 3.21 |

**Table S3**. Comparison of EL properties of iridium(III) complexes with [[Ir(ppy)_2_(N^N)] ^+^] structure, this and other work.

| Emitter: [Ir(ppy)_2_(N^N)] ^+^ | PLQY  solution  (%) | LEC Config. | λ_max,EL_ (nm) | t_1/2_ (h) | t_on max_ (h) | L_max_  (cd.m^-2^) | Efficacy_max_  (cd.A^-1^) | EQE (%) | Ref. |
| --- | --- | --- | --- | --- | --- | --- | --- | --- | --- |
| 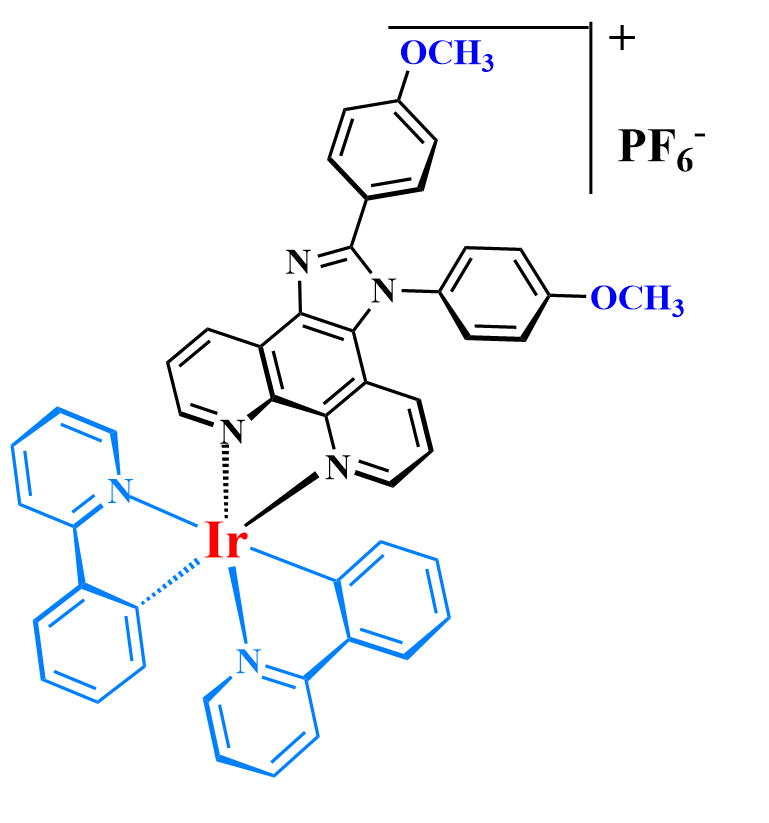 | 46 | ITO/PEDOT:PSS/Ir-iTMC:IL (4:1)/Al | 581 | 2130 | 0.65 | 870 | 8.60 | 3.1 | This work |
| 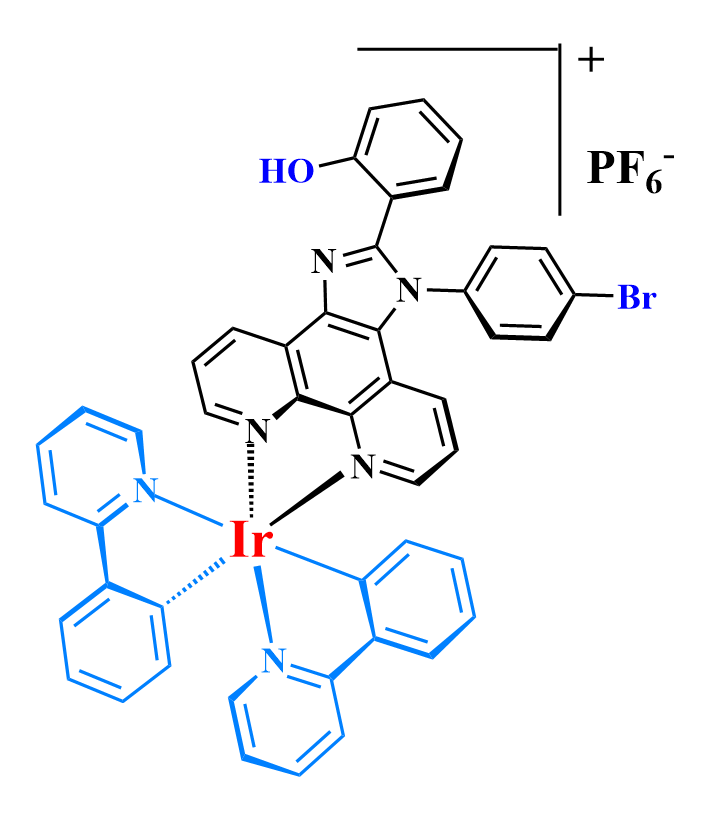 | 42 | ITO/PEDOT:PSS/Ir-iTMC:IL (4:1)/Al | 605 | 1450 | 1.30 | 563 | 5.52 | 2.5 | This work |
| 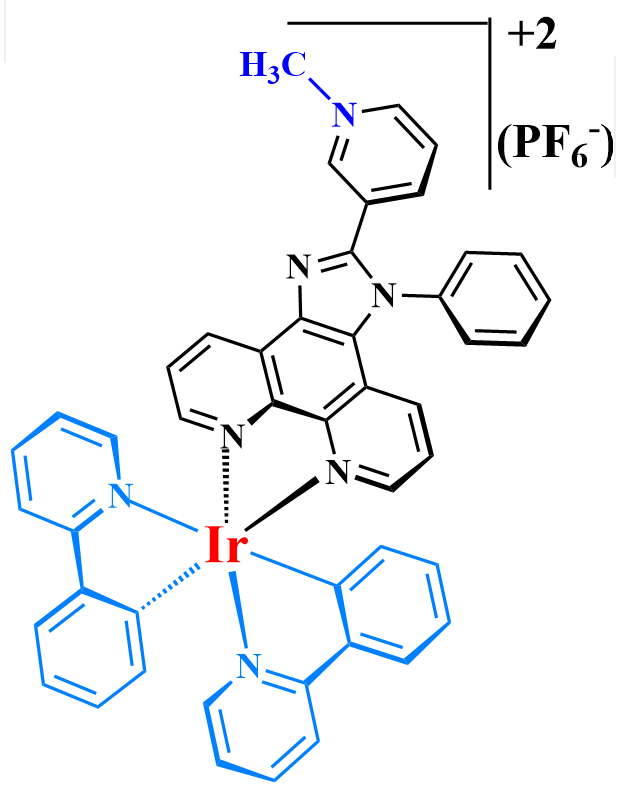 | 40 | ITO/PEDOT:PSS/Ir-iTMC:IL (4:1)/Al | 596 | 2.25 | 0.15 | 45 | 0.38 | 0.24 | This work |
| 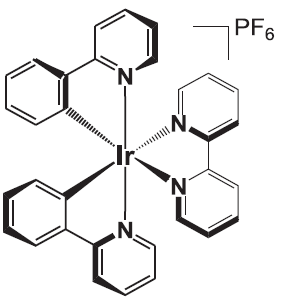 | 14 | ITO/PEDOT:PSS/Ir-iTMC:IL (4:1)/Al | 590 | 70 | 7.2 | 334 | 8.3 | 3 | [6] |
| 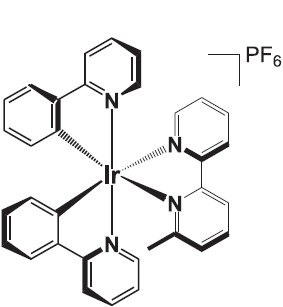 | 10 | ITO/PEDOT:PSS/Ir-iTMC:IL (4:1)/Al | 583 | 269 | 44 | 144 | 9.2 | 2.8 | [6] |
| 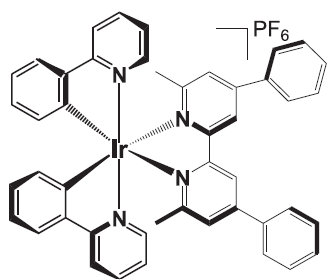 | 54 | ITO/PEDOT:PSS/Ir-iTMC:IL (4:1)/Al | 570 | 356 | 37 | 190 | 6.7 | 2.2 | [6] |
| 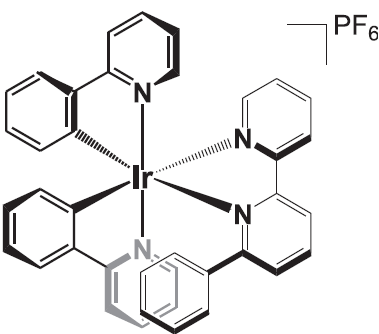 | 3 | ITO/PEDOT:PSS/Ir-iTMC:IL (4:1)/Al | 593 | 1290 | 237 | 109 | 3.1 | 1.3 | [7] |
| 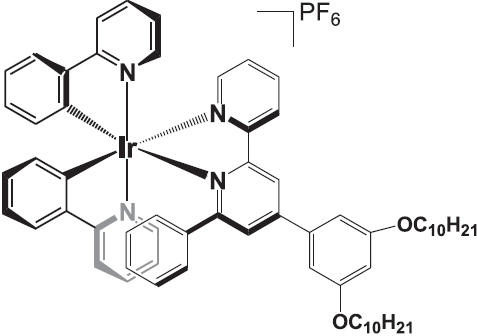 | 7 | ITO/PEDOT:PSS/Ir-iTMC:IL (4:1)/Al | 598 | 660 | 33 | 284 | 14.7 | 6.1 | [7] |
| 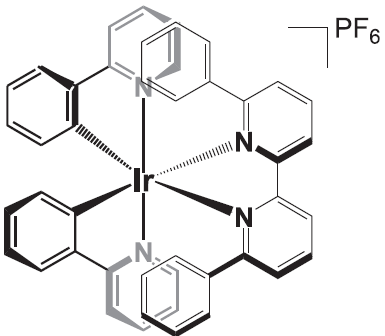 | 3 | ITO/PEDOT:PSS/Ir-iTMC:IL (4:1)/Al | 596 | 1300 | - | 70 | 2.7 | 1 | [8] |
| 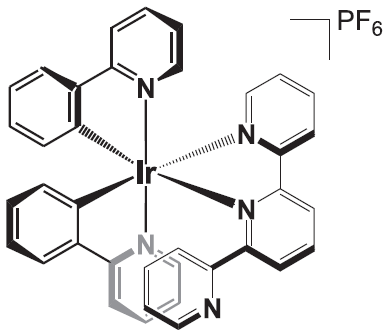 | 1.7 | ITO/PEDOT:PSS/Ir-iTMC:IL (4:1)/Al | 598 | - | .016 | 11 | - | <0.1 | [9] |
| 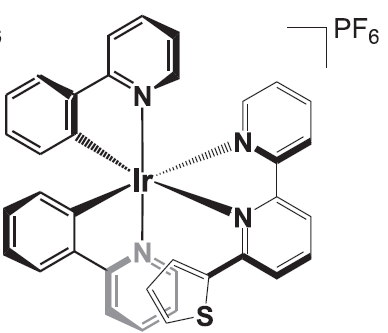 | <1 | ITO/PEDOT:PSS/Ir-iTMC:IL (4:1)/Al | 594 | 335 | 28 | 46 | 2.7 | 0.9 | [10] |
| 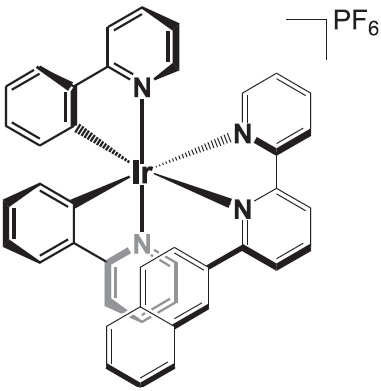 | 7.7 | ITO/PEDOT:PSS/Ir-iTMC:IL (4:1)/Al | 588 | >350 | - | 330 | 3.2 | - | [11] |
| 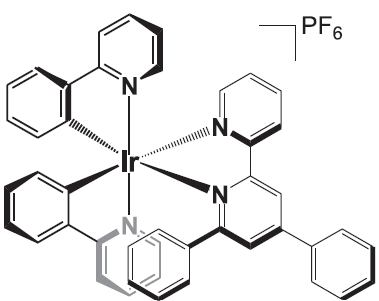 | <1 | ITO/PEDOT:PSS/Ir-iTMC:IL (4:1)/Al | 600 | 125 | 2.4 | 114 | 2.4 | 1.2 | [10] |
| 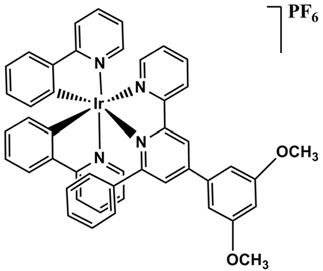 | - | ITO/PEDOT:PSS/Ir-iTMC:IL (4:1)/Al | - | 4000> | - | 670 | 3.6 | - | [12] |
| 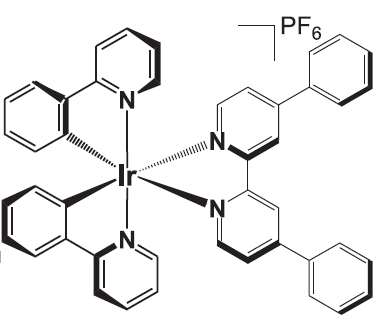 | 24.9 | ITO/PEDOT:PSS/Ir-iTMC+0.3% LiPF_6_/LiF/Al | 613 | 232 | 0.28 | 382 | 0.76 | 0.53 | [13] |
| 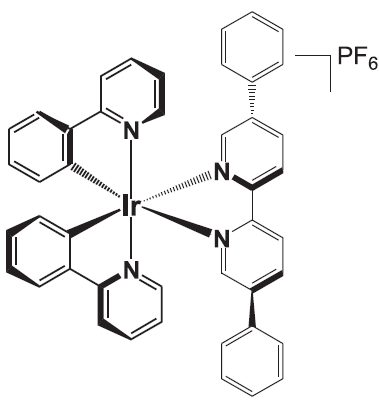 | 25.6 | ITO /Ir-iTMC + IL (0.188%)/Au | 594 | 5 min | 7 min | - | - | 0.4 | [14] |
| 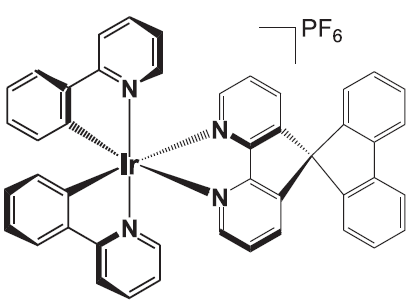 | 22.6 | ITO/Ir-iTMC:(BMIM)PF_6_/Ag | 580 | 26 | 2.8 | 330 | - | 6.2 | [15] |
| 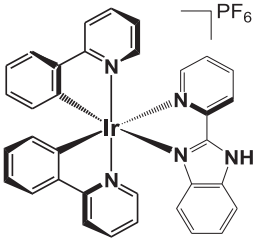 | 38.5 | ITO/PEDOT:PSS/Ir-iTMC:IL (4:1)/Al | 573 | 0.3 | 0.3 | 460 | 4.6 | 1.4 | [16] |
| 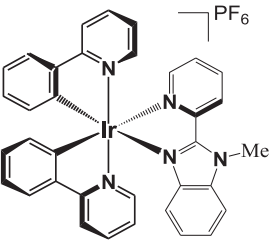 | 39.4 | ITO/PEDOT:PSS/Ir-iTMC:IL (4:1)/Al | 576 | 2700 | 28 | 904 | 9.2 | 3 | [16] |
| 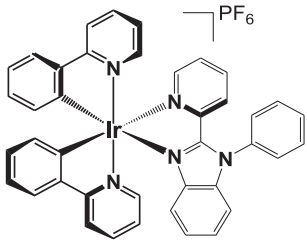 | 9 | ITO/PEDOT:PSS/Ir-iTMC:IL (1:0.45)/Al | 588 | - | 9.2 | 395 | 13.2 | 6.1 | [17] |
| 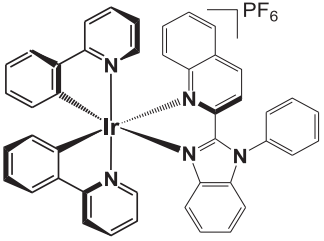 | 3 | ITO/PEDOT:PSS/Ir-iTMC:IL (1:0.45)/Al | 650 | - | 7.2 | 70 | 1.6 | 2.6 | [17] |
| 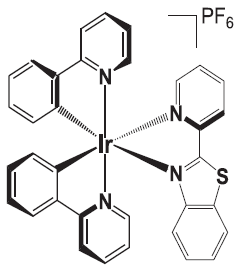 | 7.7 | ITO/PEDOT:PSS/Ir-iTMC:IL (4:1)/Al | 636 | >1000 | 54 | 77 | 0.75 | 0.7 | [18] |
| 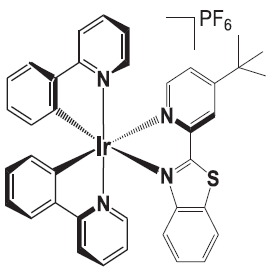 |  | ITO/PEDOT:PSS/Ir-iTMC:IL (4:1)/Al | 642 | >6000 | 870 | 200 | 2.02 | 2 | [18] |
| 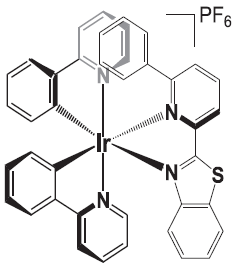 | 6.5 | ITO/PEDOT:PSS/Ir-iTMC:IL (4:1)/Al | 651 | >4500 | 63 | 119 | 1.22 | 1.49 | [18] |
| 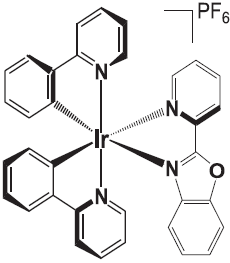 | 5.9 | ITO/PEDOT:PSS/Ir-iTMC:IL (4:1)/Al | 598 | 9 | 0.84 | 97 | 0.97 | 0.55 | [18] |
| 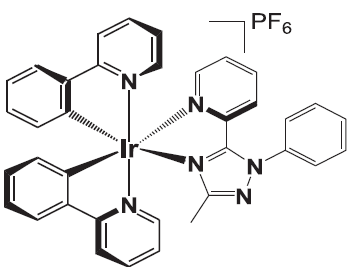 | 48 | ITO/PEDOT:PSS/Ir-iTMC:IL (5:1)/Al | 570 | 2.67 | 0.21 | 60.3 | - | - | [19] |
| 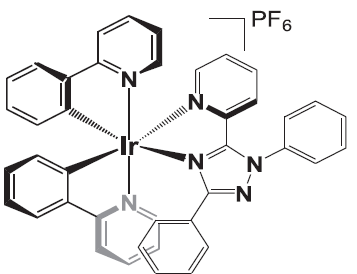 | - | ITO/PEDOT:PSS/Ir-iTMC:IL (5:1)/Al | 574 | 5.9 | 0.3 | 63.3 | - | - | [20] |
| 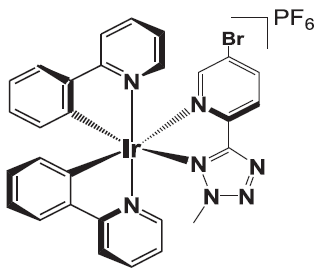 | - | ITO/PEDOT:PSS/Ir-iTMC/Al | 576 | - | - | 66 | - | - | [21] |
| 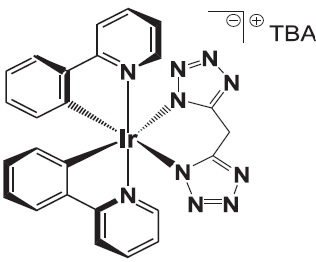 | 75 | ITO/PEDOT:PSS/Ir-iTMC:IL (4:1)/Al | 584 | >70 | 43 | 3 | 0.06 | 0.03 | [22] |
| 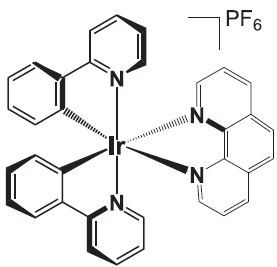 | 17 | ITO/PEDOT:PSS/Ir-iTMC:IL (4:1)/Al | 578 | 73 | 6.4 | 63 | 5.8 | 2.1 | [23] |
| 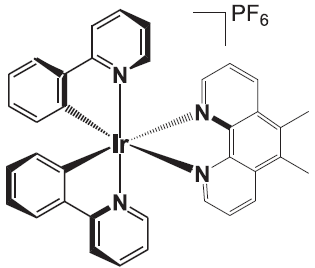 | - | ITO/PEDOT:PSS/Ir-iTMC/Al | 577 | - | - | >1200 | 2.31 | - | [24] |
| 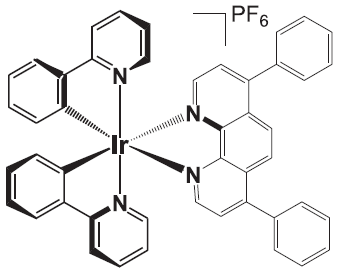 | 53 | ITO/ Ir-iTMC/Au | 600 | 65 | - | About 27 | - | - | [25] |
| 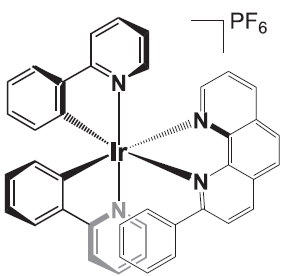 | 13 | ITO/PEDOT:PSS/Ir-iTMC:IL (4:1)/Al | 578 | 230 | 13 | 92 | 5.2 | - | [26] |
| 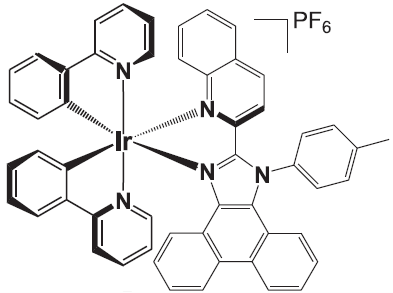 | 16 | ITO/PEDOT:PSS/Ir-iTMC/Al | 618 | - | - | 808 | 0.73 | - | [27] |
| 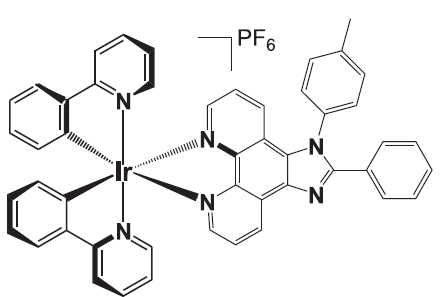 | 43 | ITO/PEDOT:PSS/Ir-iTMC:IL (4:1)/Al | 589 | 2000 | 0.47 | 689 | 6.5 | - | [28] |

# References:

1. S.-W. Hwang and Y. Chen, *Macromolecules*, 2001, **34**, 2981-2986.

2. Y. Liu, M. Liu and A. Y. Jen, *Acta polymerica*, 1999, **50**, 105-108.

3. D. Tordera, J. Frey, D. Vonlanthen, E. Constable, A. Pertegás, E. Ortí, H. J. Bolink, E. Baranoff and M. K. Nazeeruddin*, Advanced Energy Materials, 2013,* ***3,*** *1338-1343.*

4. S. Sprouse, K. King, P. Spellane and R. J. Watts, *Journal of the American chemical society*, 1984, **106**, 6647-6653.

5. L.-Y. Zhang, Y.-J. Hou, M. Pan, L. Chen, Y.-X. Zhu, S.-Y. Yin, G. Shao and C.-Y. Su, *Dalton Transactions*, 2015, **44**, 15212-15219.

6. R. D. Costa, E. Ortí, D. Tordera, A. Pertegás, H. J. Bolink, S. Graber, C. E. Housecroft, L. Sachno, M. Neuburger and E. C. Constable, *Advanced Energy Materials*, 2011, **1**, 282-290.

7. R. D. Costa, E. Ortí, H. J. Bolink, S. Graber, C. E. Housecroft and E. C. Constable, *Advanced Functional Materials*, 2010, **20**, 1511-1520.

8. R. D. Costa, E. Ortí, H. J. Bolink, S. Graber, C. E. Housecroft, M. Neuburger, S. Schaffner and E. C. Constable, *Chemical communications*, 2009, 2029-2031.

9. E. C. Constable, C. E. Housecroft, G. E. Schneider, J. A. Zampese, H. J. Bolink, A. Pertegás and C. Roldan-Carmona, *Dalton transactions*, 2014, **43**, 4653-4667.

10. E. C. Constable, C. E. Housecroft, P. Kopecky, C. J. Martin, I. A. Wright, J. A. Zampese, H. J. Bolink and A. Pertegas, *Dalton Transactions*, 2013, **42**, 8086-8103.

11. G. E. Schneider, A. Pertegás, E. C. Constable, C. E. Housecroft, N. Hostettler, C. D. Morris, J. A. Zampese, H. J. Bolink, J. M. Junquera-Hernández and E. Orti, *Journal of materials chemistry C*, 2014, **2**, 7047-7055.

12. D. Tordera, S. Meier, M. Lenes, R. D. Costa, E. Ortí, W. Sarfert and H. J. Bolink, *Advanced Materials*, 2012, **24**, 897-900.

13. K. J. Suhr, L. D. Bastatas, Y. Shen, L. A. Mitchell, G. A. Frazier, D. W. Taylor, J. D. Slinker and B. J. Holliday, *Dalton Transactions*, 2016, **45**, 17807-17823.

14. L. Sun, A. Galan, S. Ladouceur, J. D. Slinker and E. Zysman-Colman, *Journal of Materials Chemistry*, 2011, **21**, 18083-18088.

15. H. C. Su, F. C. Fang, T. Y. Hwu, H. H. Hsieh, H. F. Chen, G. H. Lee, S. M. Peng, K. T. Wong and C. C. Wu, *Advanced functional materials*, 2007, **17**, 1019-1027.

16. M. Martínez-Alonso, J. Cerdá, C. Momblona, A. Pertegás, J. M. Junquera-Hernández, A. Heras, A. M. Rodríguez, G. Espino, H. Bolink and E. Ortí, *Inorganic chemistry*, 2017, **56**, 10298-10310.

17. L. He, J. Qiao, L. Duan, G. Dong, D. Zhang, L. Wang and Y. Qiu, *Advanced Functional Materials*, 2009, **19**, 2950-2960.

18. C. D. Ertl, C. Momblona, A. Pertegás, J. M. Junquera-Hernandez, M.-G. La-Placa, A. Prescimone, E. Ortí, C. E. Housecroft, E. C. Constable and H. J. Bolink, *Journal of the American Chemical Society*, 2017, **139**, 3237-3248.

19. Q. Zeng, F. Li, T. Guo, G. Shan and Z. Su, *Scientific reports*, 2016, **6**, 1-9.

20. Q. Zeng, F. Li, T. Guo, G. Shan and Z. Su, *Organic Electronics*, 2017, **42**, 303-308.

21. Y. Kwon and Y. Choe, *Journal of Solution Chemistry*, 2014, **43**, 1710-1721.

22. E. Matteucci, A. Baschieri, A. Mazzanti, L. Sambri, J. Avila, A. Pertegas, H. J. Bolink, F. Monti, E. Leoni and N. Armaroli, *Inorganic Chemistry*, 2017, **56**, 10584-10595.

23. R. D. Costa, E. Ortí, H. J. Bolink, S. Graber, S. Schaffner, M. Neuburger, C. E. Housecroft and E. C. Constable, *Advanced functional materials*, 2009, **19**, 3456-3463.

24. S. Park, C. D. Sunesh, H. Kim, H. Chae, J. Lee and Y. Choe, *Surface and interface analysis*, 2012, **44**, 1479-1482.

25. H. J. Bolink, L. Cappelli, E. Coronado, M. Grätzel, E. Ortí, R. D. Costa, P. M. Viruela and M. K. Nazeeruddin, *Journal of the American Chemical Society*, 2006, **128**, 14786-14787.

26. R. D. Costa, E. Ortí, H. J. Bolink, S. Graber, C. E. Housecroft and E. C. Constable, *Chemical Communications*, 2011, **47**, 3207-3209.

27. J. Yeonah, C. D. Sunesh, R. K. Chitumalla, J. Jang and Y. Choe, *Organic Electronics*, 2018, **54**, 167-176.

28. D. Tordera, A. Pertegás, N. M. Shavaleev, R. Scopelliti, E. Ortí, H. J. Bolink, E. Baranoff, M. Grätzel and M. K. Nazeeruddin, *Journal of Materials Chemistry*, 2012, **22**, 19264-19268.
